# Supplementary figures and images for: Updated meta-analysis of fractional flow reserve versus coronary angiography for guiding percutaneous coronary intervention
Source: PLoS One. 2025 Oct 9;20(10):e0334019. doi: 10.1371/journal.pone.0334019 (PMC12510650; doi:10.1371/journal.pone.0334019)

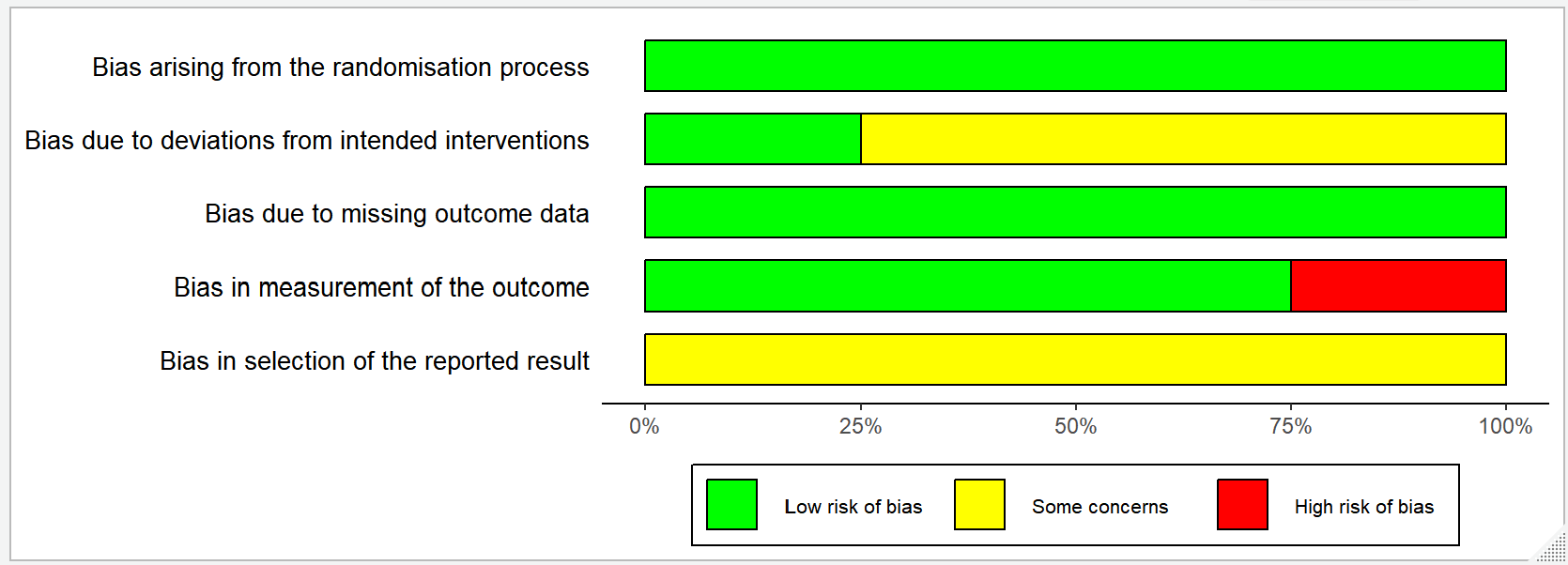

Supplement: S1 Fig — (PNG) [file pone.0334019.s001.png]

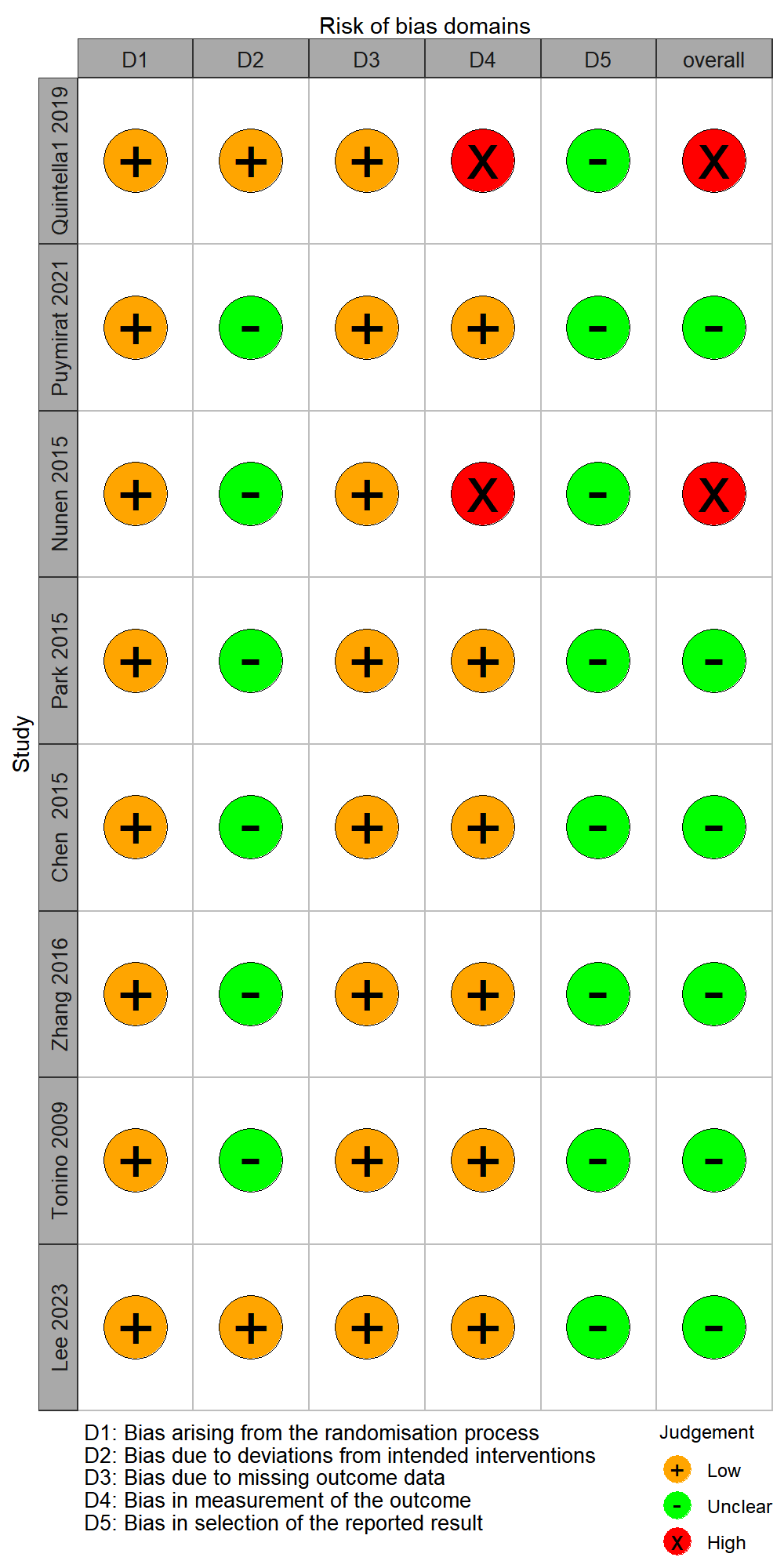

Supplement: S2 Fig — (PNG) [file pone.0334019.s002.png]
