# Supplementary material for: Updated meta-analysis of fractional flow reserve versus coronary angiography for guiding percutaneous coronary intervention
Source: PLoS One. 2025 Oct 9;20(10):e0334019. doi: 10.1371/journal.pone.0334019 (PMC12510650; doi:10.1371/journal.pone.0334019)
Supplement: S1 Appendix — (PNG) [file pone.0334019.s004.docx]

**S1 Table.** Sensitivity analysis of comparing different effect model

| Outcomes | Subgroup | Effect model | OR | 95%CI | P value | *I^2^* |
| --- | --- | --- | --- | --- | --- | --- |
| MACE | Short term | Fixed | 0.85 | 0.67-1.08 | 0.194 | 35.9% |
|  |  | Random | 0.89 | 0.65-1.23 | 0.473 |  |
|  | Long term | Fixed | 0.76 | 0.60-0.96 | 0.022 | 47.6% |
|  |  | Random | 0.71 | 0.46-1.08 | 0.110 |  |
|  | ACS group | Fixed | 0.76 | 0.53-1.08 | 0.127 | 76.9% |
|  |  | Random | 0.76 | 0.34-1.67 | 0.489 |  |
|  | Non-ACS group | Fixed | 0.82 | 0.68-0.99 | 0.038 | 0.0% |
|  |  | Random | 0.82 | 0.68-0.99 | 0.039 |  |
| All-cause mortality | Short term | Fixed | 0.77 | 0.47-1.26 | 0.296 | 0.0% |
|  |  | Random | 0.77 | 0.47-1.26 | 0.303 |  |
|  | Long term | Fixed | 0.74 | 0.50-1.09 | 0.123 | 54.8% |
|  |  | Random | 0.66 | 0.27-1.60 | 0.359 |  |
|  | ACS group | Fixed | 0.60 | 0.35-1.02 | 0.060 | 32.8 |
|  |  | Random | 0.61 | 0.31-1.19 | 0.149 |  |
|  | Non-ACS group | Fixed | 0.84 | 0.58-1.21 | 0.349 | 0.0% |
|  |  | Random | 0.84 | 0.58-1.21 | 0.352 |  |
| Myocardial infarction | Short term | Fixed | 0.85 | 0.63-1.16 | 0.307 | 39.9% |
|  |  | Random | 0.88 | 0.58-1.36 | 0.575 |  |
|  | Long term | Fixed | 0.65 | 0.45-0.93 | 0.018 | 71.9% |
|  |  | Random | 0.53 | 0.22-1.30 | 0.167 |  |
|  | ACS group | Fixed | 0.77 | 0.47-1.25 | 0.294 | 77.1% |
|  |  | Random | 0.74 | 0.24-2.28 | 0.602 |  |
|  | Non-ACS group | Fixed | 0.76 | 0.58-0.99 | 0.039 | 0.0% |
|  |  | Random | 0.76 | 0.58-0.99 | 0.040 |  |
| Revascularization | Short term | Fixed | 0.77 | 0.53-1.11 | 0.158 | 46.7% |
|  |  | Random | 0.84 | 0.46-1.53 | 0.574 |  |
|  | Long term | Fixed | 0.84 | 0.64-1.11 | 0.215 | 0.0% |
|  |  | Random | 0.84 | 0.64-1.11 | 0.222 |  |
|  | ACS group | Fixed | 0.98 | 0.48-2.02 | 0.958 | 71.3% |
|  |  | Random | 1.13 | 0.25-5.15 | 0.873 |  |
|  | Non-ACS group | Fixed | 0.80 | 0.63-1.01 | 0.055 | 0.0% |
|  |  | Random | 0.80 | 0.63-1.01 | 0.057 |  |
