# Supplementary material for: Updated meta-analysis of fractional flow reserve versus coronary angiography for guiding percutaneous coronary intervention
Source: PLoS One. 2025 Oct 9;20(10):e0334019. doi: 10.1371/journal.pone.0334019 (PMC12510650; doi:10.1371/journal.pone.0334019)
Supplement: Attachment — Submitted filename: PONE-D-24-28817_R2.pdf [file pone.0334019.s010.pdf]

PLOS ONE

Updated Meta-Analysis of Fractional Flow Reserve versus Coronary Angiography for Guiding Percutaneous Coronary Intervention.

--Manuscript Draft--

Manuscript Number: PONE-D-24-28817R2

Article Type: Research Article

Full Title: Updated Meta-Analysis of Fractional Flow Reserve versus Coronary Angiography for Guiding Percutaneous Coronary Intervention.

Short Title: Meta-analysis of FFR versus CAG for guiding PCI

Corresponding Author: fahui yin
Wuwei Liangzhou Hospital
Wuwei, China CHINA

Keywords: Fractional Flow Reserve , Coronary Angiography , Percutaneous Coronary Intervention

Abstract:

Abstract

Background: Fractional Flow Reserve (FFR) has been widely used in clinical practice for decades; however, the comparative clinical outcomes of FFR-guided versus coronary angiography (CAG)-guided percutaneous coronary intervention (PCI) require further comprehensive evaluation.

Method: Randomized controlled trials (RCTs) comparing FFR-guided and CAG-guided PCI were systematically searched across PubMed, Embase and the Cochrane library databases from their inception to December 31, 2023. The primary endpoints included the incidence of major adverse cardiovascular events (MACE), all cause mortality, myocardial infarction (MI) and target vessel revascularization(TVR). Stratified analyses were conducted to evaluate the effects of FFR-guided versus CAG-guided PCI across different follow-up peri[illegible]hort-term and long-term) and patient categories (ACS and non-ACS patients).

Results: This meta-analysis included 8 RCTs involving 4,433 patients, with 4 studies reporting 1-year outcomes and 4 reporting outcomes beyond 1 year. Among these, 5 studies focused on non-ACS patients, and 3 included ACS patients, with a significant male predominance (3,437 vs. 996 females). By follow-up duration, FFR-guided PCI demonstrated significant long-term reductions in MACE (OR: 0.76, 95% CI: 0.60-0.96, P = 0.022) and MI (OR: 0.65, 95% CI: 0.45-0.93, P = 0.018), but no significant short-term benefits were observed for MACE (OR: 0.85, 95% CI: 0.67-1.08, P = 0.194), MI (OR: 0.85, 95% CI: 0.63-1.16, P = 0.307), or all-cause mortality (short-term: OR: 0.77, 95% CI: 0.47-1.26, P = 0.296; long-term: OR: 0.74, 95% CI: 0.50-1.09, P = 0.123). By patient type, FFR-guided PCI significantly reduced MACE (OR: 0.82, 95% CI: 0.68-0.99, P = 0.038), MI (OR: 0.76, 95% CI: 0.58-0.99, P = 0.039), and TVR (OR: 0.78, 95% CI: 0.61-0.99, P = 0.036) in non-ACS patients, but no significant differences were observed in ACS patients for MACE (OR: 0.76, 95% CI: 0.53-1.08, P = 0.127), all-cause mortality (OR: 0.60, 95% CI: 0.35-1.02, P = 0.060), MI (OR: 0.77, 95% CI: 0.47-1.25, P = 0.294), or TVR (OR: 0.98, 95% CI: 0.48-2.02, P = 0.315). Sensitivity analysis confirmed the robustness of these findings.

Conclusions: FFR-guided PCI is superior to CAG-guided PCI in reducing MACE and MI in long-term and non-ACS patients but shows no advantage in short-term or ACS populations. Future research endeavors should focus on optimizing the application of FFR in ACS patients to potentially enhance clinical outcomes.

Order of Authors:
fahui yin
Yong Zhang
Xueqian Zhang
Yangang Chen
Xuelian Cui

Response to Reviewers:

Dear Reviewer#4

Thank you very much for your thoughtful and constructive feedback on our manuscript. We greatly appreciate the time and effort you have dedicated to reviewing our work

	and providing valuable insights.<br>Regarding your comment about the potential overlap between the patient populations in Nunen et al. (2015) and Tonino et al. (2009), we fully agree with your observation that both studies derive from the FAME study population. To address this concern and avoid double-counting, we have carefully revised our analysis approach. Specifically, we have refrained from pooling the data from these two studies in the meta-analysis. Instead, we conducted a stratified subgroup analysis to ensure that the FAME study population is not redundantly included. This approach allows us to maintain the integrity of our findings while respecting the methodological considerations you raised. Furthermore, we have explicitly acknowledged this limitation in the Limitations section of our manuscript, emphasizing our efforts to mitigate potential biases arising from overlapping datasets. We believe this adjustment strengthens the robustness of our analysis and aligns with your recommendation.<br>Once again, we sincerely appreciate your attention to this important detail. Your feedback has significantly improved the quality of our work. We hope that our revised approach and clarification adequately address your concern. Please do not hesitate to let us know if there are any additional points you would like us to consider.<br>Thank you for your time and expertise.<br>Best regards,<br>Fahui Yin<br>cardiovascular department， Wuwei City Liangzhou hospital<br>414380580@qq.com
**Additional Information:**	
**Question**	**Response**
**Financial Disclosure**<br><br>Enter a financial disclosure statement that describes the sources of funding for the work included in this submission. Review the submission guidelines for detailed requirements. View published research articles from *PLOS ONE* for specific examples.<br><br>This statement is required for submission and **will appear in the published article** if the submission is accepted. Please make sure it is accurate.<br><br>**Funded studies**<br>Enter a statement with the following details:<br>• Initials of the authors who received each award<br>• Grant numbers awarded to each author<br>• The full name of each funder<br>• URL of each funder website<br>• Did the sponsors or funders play any role in the study design, data collection and analysis, decision to publish, or preparation of the manuscript?<br><br>Did you receive funding for this work?	The author(s) received no specific funding for this work.

**Competing Interests**<br><br>Use the instructions below to enter a competing interest statement for this submission. On behalf of all authors, disclose any competing interests that could be perceived to bias this work—acknowledging all financial support and any other relevant financial or non-financial competing interests.<br><br>This statement is required for submission and **will appear in the published article** if the submission is accepted. Please make sure it is accurate and that any funding sources listed in your Funding Information later in the submission form are also declared in your Financial Disclosure statement.<br><br>View published research articles from *PLOS ONE* for specific examples.<br><br>**NO authors have competing interests**<br><br>Enter: *The authors have declared that no competing interests exist.*<br><br>**Authors with competing interests**<br><br>Enter competing interest details beginning with this statement:<br><br>*I have read the journal's policy and the authors of this manuscript have the following competing interests: [insert competing interests here]*<br><br>* typeset	NO authors have competing interests
**Ethics Statement**<br><br>Enter an ethics statement for this submission. This statement is required if the study involved:<br><br>• Human participants<br>• Human specimens or tissue<br>• Vertebrate animals or cephalopods	N/A

• Vertebrate embryos or tissues<br>• Field research<br><br>Write "N/A" if the submission does not require an ethics statement.<br><br>General guidance is provided below. Consult the submission guidelines for detailed instructions. **Make sure that all information entered here is included in the Methods section of the manuscript.**	

Powered by Editorial Manager® and ProduXion Manager® from Aries Systems Corporation

**Format for specific study types**<br><br>**Human Subject Research (involving human participants and/or tissue)**<br>• Give the name of the institutional review board or ethics committee that approved the study<br>• Include the approval number and/or a statement indicating approval of this research<br>• Indicate the form of consent obtained (written/oral) or the reason that consent was not obtained (e.g. the data were analyzed anonymously)<br><br>**Animal Research (involving vertebrate animals, embryos or tissues)**<br>• Provide the name of the Institutional Animal Care and Use Committee (IACUC) or other relevant ethics board that reviewed the study protocol, and indicate whether they approved this research or granted a formal waiver of ethical approval<br>• Include an approval number if one was obtained<br>• If the study involved *non-human primates*, add *additional details* about animal welfare and steps taken to ameliorate suffering<br>• If anesthesia, euthanasia, or any kind of animal sacrifice is part of the study, include briefly which substances and/or methods were applied<br><br>**Field Research**<br><br>Include the following details if this study involves the collection of plant, animal, or other materials from a natural setting:<br>• Field permit number<br>• Name of the institution or relevant body that granted permission	
**Data Availability**<br><br>Authors are required to make all data underlying the findings described fully available, without restriction, and from the time of publication. PLOS allows rare exceptions to address legal and ethical concerns. See the PLOS Data Policy and FAQ for detailed information.	Yes - all data are fully available without restriction

Powered by Editorial Manager® and ProduXion Manager® from Aries Systems Corporation

A Data Availability Statement describing where the data can be found is required at submission. Your answers to this question constitute the Data Availability Statement and **will be published in the article**, if accepted.<br><br>**Important:** Stating 'data available on request from the author' is not sufficient. If your data are only available upon request, select 'No' for the first question and explain your exceptional situation in the text box.<br><br>Do the authors confirm that all data underlying the findings described in their manuscript are fully available without restriction?	
**Describe where the data may be found in full sentences. If you are copying our sample text, replace any instances of XXX with the appropriate details.**<br><br>• If the data are **held or will be held in a public repository**, include URLs, accession numbers or DOIs. If this information will only be available after acceptance, indicate this by ticking the box below. For example: *All XXX files are available from the XXX database (accession number(s) XXX, XXX.)*.<br>• If the data are all contained **within the manuscript and/or Supporting Information files**, enter the following: *All relevant data are within the manuscript and its Supporting Information files.*<br>• If neither of these applies but you are able to provide **details of access elsewhere**, with or without limitations, please do so. For example:<br><br>*Data cannot be shared publicly because of [XXX]. Data are available from the XXX Institutional Data Access / Ethics Committee (contact via XXX) for researchers who meet the criteria for access to confidential data.*<br><br>*The data underlying the results presented in the study are available from (include the name of the third party*	All relevant data are within the manuscript and its Supporting Information files.

and contact information or URL).<br>• This text is appropriate if the data are owned by a third party and authors do not have permission to share the data.<br><br>* typeset	
Additional data availability information:	

Manuscript Click here to access/download;Manuscript;Manuscript.pdf

Title Page

Manuscript title: Updated Meta-Analysis of Fractional Flow Reserve versus Coronary Angiography for Guiding Percutaneous Coronary Intervention.

Short title: Meta-analysis of FFR versus CAG guided PCI.

Author list: Fahui Yin[1*]MD; Yong Zhang[1] MD; Xueqian Zhang[1]; Yangang Chen[1]MD; Xuelian Cui[1];

1 department of cardiovascular disease, Liangzhou Hospital, Wuwei City, Gansu Province, China

***Corresponding author:** Fahui Yin, MD, department of cardiovascular disease, Liangzhou Hospital, Wuli new village, Gaoba Town, Liangzhou District, Wuwei City, Gansu Province, China.

E-mail: 414380580@qq.com.

Co-author: Yong Zhang[1] MD department of cardiovascular disease, Liangzhou Hospital, Wuli new village, Gaoba Town, Liangzhou District, Wuwei City, Gansu Province, China.

E-mail: zy8152@163.com.

Word counts: 2914 words.

Updated Meta-Analysis of Fractional Flow Reserve versus Coronary Angiography for Guiding Percutaneous Coronary Intervention

Fahui Yin MD; Yong Zhang MD; Xueqian Zhang; Yangang Chen MD; Xuelian Cui;

Abstract

Background:
Fractional Flow Reserve (FFR) has been widely used in clinical practice for decades; however, the comparative clinical outcomes of FFR-guided versus coronary angiography (CAG)-guided percutaneous coronary intervention (PCI) require further comprehensive evaluation.

Method:
Randomized controlled trials (RCTs) comparing FFR-guided and CAG-guided PCI were systematically searched across PubMed, Embase and the Cochrane library databases from their inception to December 31, 2023. The primary endpoints included the incidence of major adverse cardiovascular events (MACE), all cause mortality, myocardial infarction (MI) and target vessel revascularization(TVR). Stratified analyses were conducted to evaluate the effects of FFR-guided versus CAG-guided PCI across different follow-up periods (short-term and long-term) and patient categories (ACS and non-ACS patients).

Results:
This meta-analysis included 8 RCTs involving 4,433 patients, with 4 studies reporting 1-year outcomes and 4 reporting outcomes beyond 1 year. Among these, 5 studies focused on non-ACS patients, and 3 included ACS patients, with a significant male predominance (3,437 vs. 996 females). By follow-up duration, FFR-guided PCI demonstrated significant long-term reductions in MACE (OR: 0.76, 95% CI: 0.60-0.96, P = 0.022) and MI (OR: 0.65, 95% CI: 0.45-0.93, P = 0.018), but no significant short-term benefits were observed for MACE (OR: 0.85, 95% CI: 0.67-1.08, P = 0.194), MI (OR: 0.85, 95% CI: 0.63-1.16, P = 0.307), or all-cause mortality (short-term: OR: 0.77, 95% CI: 0.47-1.26, P = 0.296; long-term: OR: 0.74, 95% CI: 0.50-1.09, P = 0.123). By patient type, FFR-guided PCI significantly reduced MACE (OR: 0.82, 95% CI: 0.68-0.99, P = 0.038), MI (OR: 0.76, 95% CI: 0.58-0.99, P = 0.039), and TVR (OR: 0.78, 95% CI: 0.61-0.99, P = 0.036) in non-ACS patients, but no significant differences were observed in ACS patients for MACE (OR:

0.76, 95% CI: 0.53-1.08, P = 0.127), all-cause mortality (OR: 0.60, 95% CI: 0.35-1.02, P = 0.060), MI (OR: 0.77, 95% CI: 0.47-1.25, P = 0.294), or TVR (OR: 0.98, 95% CI: 0.48-2.02, P = 0.315). Sensitivity analysis confirmed the robustness of these findings.

Conclusions: FFR-guided PCI is superior to CAG-guided PCI in reducing MACE and MI in long-term and non-ACS patients but shows no advantage in short-term or ACS populations. Future research endeavors should focus on optimizing the application of FFR in ACS patients to potentially enhance clinical outcomes.

Introduction

Coronary artery disease (CAD) has been the leading cause of death worldwidely and exerts a severe health burden on human being internationally[1]. As an “golden standard” for diagnosis of CAD, coronary angiography has always been the most important method for guiding PCI. However, owing to its nature of approximation, usually over-estimates or under-estimates the severity of the intermediate coronary stenosis, and the inability to determine the hemodynamic significance of coronary stenosis. Hence, the clinical importance of anatomical stenosis based on coronary angiography challe[illegible] by FFR which could scientifically measure whether the target vessel have inducible ischemia through a special pressure guidewire[2].

FFR is defined as the ratio of the pressure distal to stenosis and the aortic pressure in a condition of maximum myocardium hyperemia. With a special coronary pressure wire, FFR could provide a readily available, straightforward and quantitative technique to evaluate the physiologic significance of a coronary lesion. When the value of FFR greater than 0.8, the stenosis is considered non-significant, and the value less than 0.75 is regarded as coronary ischemia[3]. FFR was recommended as Class IA for guiding percutaneous coronary revascularisation by European Society of Cardiology and class IIA by American Heart Association practice guideline[4,5].

Many studies have investigated the clinical outcomes of FFR-guided and CAG-guided PCI in patients with CAD. Fraction Flow Reserve Versus Angiography for Multivessel Evaluation (FAME) study showed FFR-guided PCI had lower rates of mortality, re-infarction and MACEs when compared to the standard angiography-guided PCI [6]. However, the DEFER-DES (Fractional Flow Reserve–Guided Versus Routine Drug-Eluting Stent Implantation in Patients With Intermediate Coronary Stenosis) trail, demonstrated that no significant difference was observed in cardiac death, revascularization and MACEs between the two groups

with a five-year follow up[7]. Additionally，although previous meta-ana[illegible] have compared the two strategies, most of them enrolled the observational studies and the patients with different operation type (while some patients undergone PCI, others might select coronary artery bypass graft (CABG))[8,9]. Consequently, we again compare the clinical outcomes of FFR-guided and CAG-guided PCI through even more rigorous quality control and patients selection.

Methods

Data sources and search strategy

PubMed、Embase and the Cochrane library data bases were searched to identify articles comparing CAG and FFR-guided PCI before December 31,2023. All articles were restricted to English. According to the “PICOS” principle of evidence-based medicine, the following medical subject headings and search terms were utilized: (1) fractional flow reserve; (2) coronary angiography; (3) percutaneous coronary intervention. Additionally, references of relevant articles and conference reports were screened to uncover potential suitable studies. study was carried out in accordance with the Preferred Reporting Items for Systematic Reviews and Meta-Analyses (PRISMA) statement.

Inclusion and Exclusion criteria

Studies were eligible for inclusion included if they met the following criteria: (1) Randomised controlled trails (RCTs) ; (2) Comparing FFR-guided and angiography-guided PCI; (3) Reporting at least one of the following outcomes, including MACEs, all-cause mortality, MI, and TVR. Studies were excluded if they were: (1) Non-RCTs; (2) Studies included the patients underwent CABG; (3) Did not report relevant outcomes; (4) Did not allow for the extraction of detailed data.

Subgroup analysis

In this meta-analysis, subgroup analysis were conducted based on the follow-up period and patients categories. Regarding the follow-up period, the studies were divided into short term subgroup (≤1 year) and long term follow-up subgroup(>1 year). In terms of patients categories, the studies were divided into acute coronary syndrome (ACS) subgroup and non-ACS subgroup.This type of subgroup analysis not only detect the potential heterogeneity among different studies, but also allow for a better comparison of the outcomes of FFR-guided and CAG-guided PCI in different patient populations and clinical backgrounds.

Data collection and risk of bias assessment

Firstly, all records were imported into the Endnote software（version X7, Thomson Reuters, U.S）to perform automatic duplication removal during the initial search. After eliminating duplicates, three reviewers (Yin FH, Zhang Y, and Zhang [illegible] independently screened the titles and abstracts and excluded the irrelevant records. Subsequently, the full-text articles of the remaining records were retrieved for in-depth screening. Additionally, the reference of relevant articles and conference reports were also screened for potential suitable studies. A standard data extraction form was was designed prior to data extraction. From each included study, the following information was extracted: the first author, year of publication, nationality, follow-up duration, sample size of each study, baseline characteristics, and the clinical outc[illegible].The Revised Cochrane risk of bias tool (ROB2) was utilized to assess the quality of each study[10]. The quality evaluation of each study was also independently cond[illegible] Any discrepancy in data extraction and quality assessment were resolved through consensus.

Statistical analysis

Continuous variables were expressed as mean ± standard deviation (SD), while categorical variables were presented as numbers and percentages. Statistical analyses were performed using Stata software (version 18.0, Stata Corp, College Station, Texas). Heterogeneity among the included studies was assessed using the Chi-square test and quantified by the I^2 statistic. Given that all outcomes in this study were dichotomous data, they were pooled and expressed as odds ratios (ORs). A fixed-effect model (Mantel-Haenszel method) was applied when $I^2 < 50\%$, indicating low heterogeneity, whereas a random-effect model (DerSimonian-Laird method) was used for $I^2 \geqslant 50\%$, indicating significant heterogeneity, with greater weight assigned to smaller studies. Subgroup analyses were conducted based on follow-up duration and patient categories to explore potential sources of heterogeneity and to compare fractional flow reserve (FFR) and coronary angiography

(CAG) under different clinical scenarios. Sensitivity analysis was performed by comparing fixed- and random-effect models. Due to the limited number of included studies (fewer than ten), funnel plots were not utilized, as their power to detect asymmetry would be insufficient. Results were reported as ORs with 95% confidence intervals (CIs), and a p-value < 0.05 was considered statistically significant.

Results

Searching results and quality assessment

A comprehensive literature search was conducted in PubMed, Embase, and the Cochrane Library from their inception to December 31, 2023.The initial literature searching yielded 1959 articles, which were independently screened by three team members (Yin FH, Zhang Y, Zhang XQ) . An additional 9 articles were identified through manual reference checking of the included studies. First, duplicates were removed through automated and manual deduplication processes. Second, after screening titles and abstracts, 29 articles were retained by excluding irrelevant ones (as detailed in S1 Table).. Then, full text articles were obtained and carefully reviewed against predefined inclusion and exclusion c[illegible]. Ultimately, 8 studies met inclusion criteria and were enrolled into this meta-analysis[6,11-17]. The study selection process is illustrated in **Fig 1**. The revised Cochrane risk of bias (Rob2) tool was utilized to assess the quality of enrolled RCTs. Most studies described the detailed process of random sequence generation. However, since blinding of participants and intervention providers was not feasible, potential deviations from the intended interventions due to the experimental context were likely, though difficult to ascertain from the articles. Six studies employed independent clinical data and events committees to evaluate endpoints in a blinded manner. All studies provided detailed clinical endpoint events and accounted for missing data, but the source of these data (whether from multiple eligible outcome measurements) was unclear. The results of the risk of bias assessment are presented in **S1 and S2 Figs.**

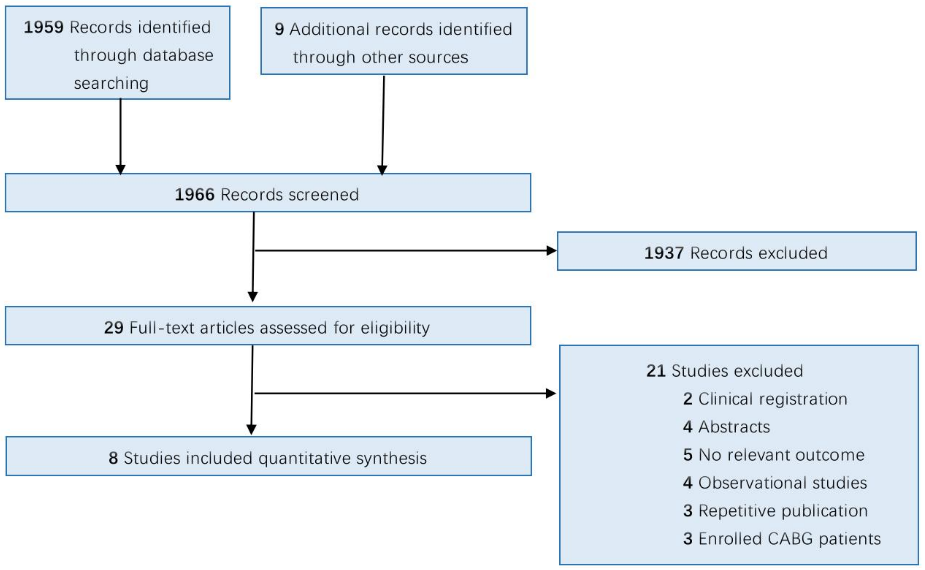

Fig 1. Flow Diagram of studies selection process.

Baseline characteristics of the included studies

Eight studies involving 4,433 patients that met our predefined inclusion criteria were included into this meta-analysis [6,11-17]. Among these, three studies compared FFR-guided and CAG-guided PCI in patients with multivessel patients [6,14,15]. two enrolled patients withSTEMI and multivessel patients [13,17], one focused on with older patients with NSTEMI [11], and the other two recruited patients with intermediate coronary stenosis and true coronary bifurcation lesions, respectively [7,12]. Baseline characteristics of the studies are presented in Table 1. The follow up duration was ranged from 1 year to 5 years, and the mean age ranged from 59.5 to 70 years. Male patients were significantly outnumbered female patients in overall population (3437 versus 996, respectively), with 24.5% having diabetes, 57.9% having hypertension, 56.3% having dyslipidemia, and 32.8% being current smokers. No significant difference was obsered between the two groups. Based on follow-up duration, the studies were further divided into short term (≤1 year) and long term (>1 year) follow-up groups: fourstudies with one-year follow-up belonged to the short term subgroup [6,11-13] , while the other four belonged to long term subgroup [14-17]. Aditionally, based on patients categories, the studies were divided into ACS [11,13,17] and non-ACS subgroups [6,12,14-16].

Table1. Baseline characteristics of the included studies

**Study**	**Quintella[14] 2019**		**Puymirat[13] 2021**		**Nunen[15] 2015**		**Park[16] 2015**	
Follow-up (year)	1.5		1		5		5	
Nationality*	Brazil		France		Netherland		Korea	
Type of patients	MVD		STEMI and MVD		MVD		ICS	
Group	CAG	FFR	CAG	FFR	CAG	FFR	CAG	FFR
Number of patients	35	34	577	586	429	436	115	114
Age, mean (SD),y	60± 9	63 ± 8	62 ± 11	63 ± 11	64± 10	65 ± 10	63 ± 10	62± 10
Male patients,No.(%)	22 (63)	25 (74)	468 (81)	498 (85)	318 (74)	328 (75)	83(73)	87(76)
Diabetes Mellitus,No.(%)	12 (34)	12 (35)	82(14)	107(18)	107 (25)	98 (22)	39 (34)	30 (26)
Hypertension, No.(%)	26 (74)	25 (74)	262(45)	253(43)	277 (65)	259 (59)	73 (64)	65 (57)
Dyslipedemia, No.(%)	26 (74)	24 (71)	237(41)	232(39)	316 (74)	307 (70)	78 (68)	80 (70)
Current smoker, No.(%)	9 (26)	10 (29)	210(36)	235(40)	130 (30)	111 (25)	38 (33)	30 (26)
Previous MI, No.(%)	7 (20)	8 (24)	31(5)	45(8)	155 (36)	154 (35)	20 (17)	22 (19)
Outcomes†	①②④		①②③④		①②③④		①④	
**Study**	**Chen[12] 2015**		**Zhang[11] 2016**		**Tonino[6] 2009**		**Lee[17] 2023**	
Follow-up (year)	1		1		1		3.5	
Nationality*	China		China		Netherland		Korea	
Type of patients	CAD with TCBL		NSTEMI		MVD		STEMI and MVD	
Group	CAG	FFR	CAG	FFR	CAG	FFR	CAG	FFR
Number of patients	160	160	110	110	496	509	278	284
Age, mean (SD),y	65 ± 9	65 ± 9	70 ± 3	70 ± 4	64 ± 10	65 ± 10	63 ± 12	64 ± 11
Male patients, No.(%)	116 (73)	121 (76)	78 (71)	75 (68)	360 (73)	384 (75)	234(84)	240(85)
Diabetes Mellitus,No.(%)	43 (27)	48 (30)	36 (33)	40 (36)	125 (25)	123 (24)	86(31)	97(34)
Hypertension, No.(%)	106 (68)	116 (73)	83 (76)	81 (74)	327 (66)	312 (61)	152(55)	151(53)
Dyslipedemia, No.(%)	32 (20)	27 (17)	93 (85)	90 (82)	362 (73)	366 (72)	107(39)	121(43)
Current smoker, No.(%)	64 (40)	66 (41)	31 (28)	29 (26)	156 (32)	138 (27)	105(38)	91(32)
Previous MI, No.(%)	19 (12)	12 (8)	24 (22)	23 (21)	180 (36)	187 (37)	7(3)	7(3)
Outcomes†	①②③④		①②③		①②③④		①②③④	

Values are presented as percentage; "*" indicates the first author's nationality; "†" four outcomes were included in this meta-analysis: ① indicates MACE; ②indicates all cause mortality; ③ indicates myocardial infarction; ④ targeted vessel revascularization. MVD = multi-vessel disease; STEMI = ST segment elevation myocardial infarction; ICS = intermediate coronary stenosis; CAD = coronary artery disease; TCBL = true coronary bifurcation lesion.

Clinical outcomes of FFR-guided and CAG-guided PCI

MACE. A total of 8 studies involving 4433 patients reported MACE rates, with 4 studies providing 1-year outcomes[6,11-13] and the remaining 4 reporting outcomes beyond 1 year[14-17]. Stratified analysis based on follow up duration revealed no significant difference in short-term outcomes between the FFR and CAG groups (OR: 0.85, 95%CI 0.67-1.08, P = 0.194, I^2 = 35.9%). Sensitivity analysis confirmed the robustness of the results, as the pooled effect sizes calculated using both fixed-effect and random-effects models remained consistent. However, FFR-guided PCI demonstrated significant long-term benefits in reducing MACE rates compared to CAG-guided PCI (OR:0.76, 95%CI 0.60- 0.96, P = 0.022, I^2 = 47.6%)(**Fig 2. A**). Among the included studies, 5 focused on non-ACS patients [6,12,14-16], while 3 included ACS patients [11,13,17]. Subgroup analysis by patient category showed that FFR-guided PCI was associated with greater reductions in MACE rates in the non-ACS subgroup(OR:0.82, 95%CI 0.68- 0.99, P = 0.038, I^2 = 0.0%). no significant difference was observed in the ACS subgroup (OR:0.76, 95%CI 0.53- 1.08, P = 0.127, I^2 = 76.9%)(**Fig 3. A**).

All-cause Mortality. When assessing all-cause mortality, 7 studies involving 4204 patients reported relevant data [6,11-15,17], including 4 studies with 1-year outcomes [6,11-13] and 3 studies with outcomes beyond 1 year [14,15,17]. Stratified analysis by follow-up duration revealed no significant differences in either the short-term (OR: 0.77, 95% CI: 0.47-1.26, P = 0.296, I^2 = 0%) or long-term groups (OR: 0.74, 95% CI: 0.50-1.09, P = 0.123, I^2 = 54.8%) (**Fig 2.B**).Further stratification by patient category showed that 4 studies focused on non-ACS patients [6,12,14,15], while 3 studies included ACS patients [11,13,17]. Subgroup analysis demonstrated no significant differences in all-cause mortality between FFR-guided and CAG-guided PCI, both in the ACS subgroup (OR: 0.60, 95% CI: 0.35-1.02, P = 0.060, I^2 = 32.8%) and the non-ACS subgroup (OR: 0.84, 95% CI: 0.58-1.21, P = 0.349, I^2 = 0%)(**Fig 3. B**). Sensitivity analysis confirmed the consistency of these findings, as the pooled effect sizes calculated using both fixed-effect and random-effects

models remained stable across subgroups.

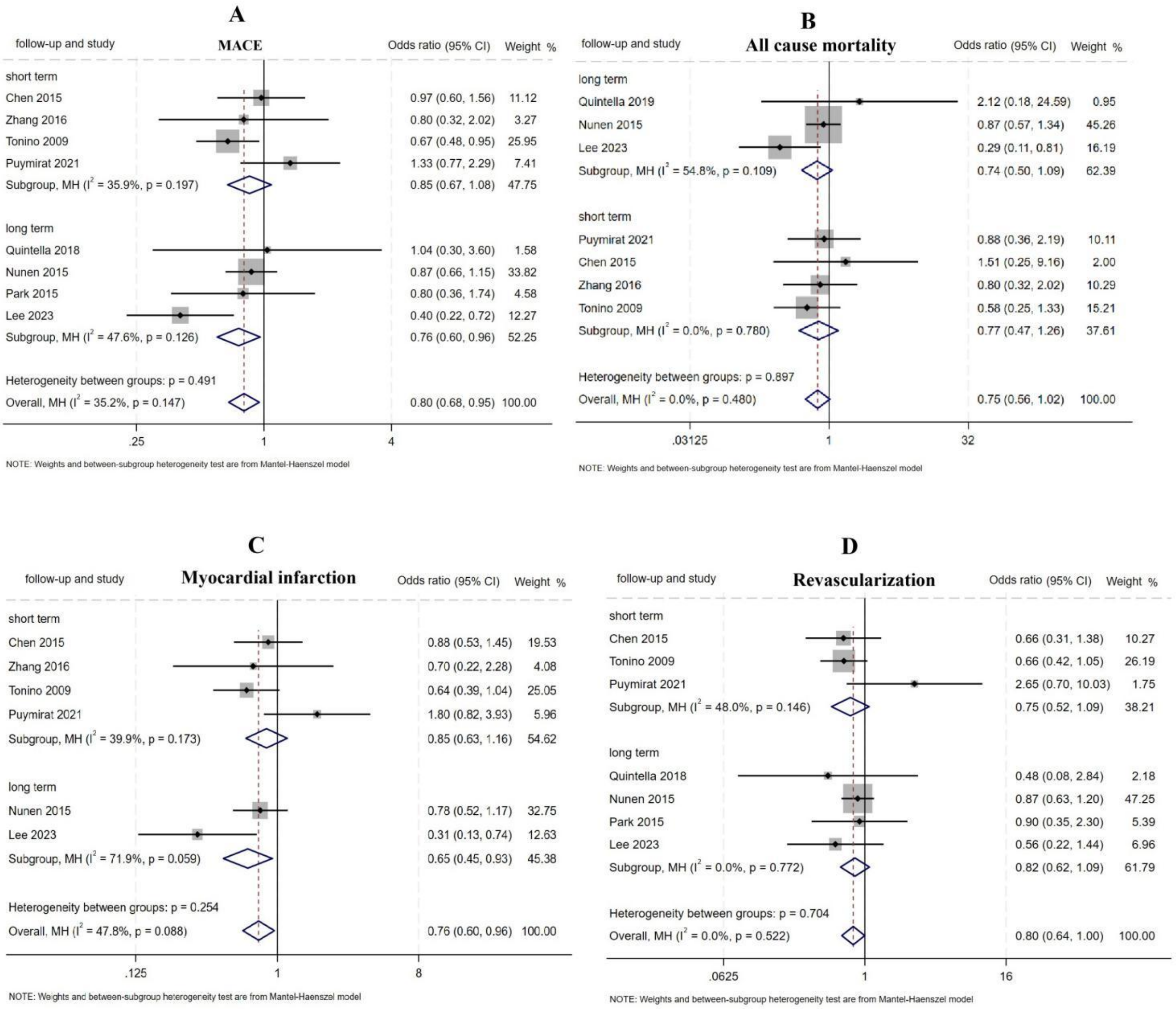

Fig 2. Forest plot of subgroup analysis comparing FFR-guided versus CAG-guided PCI stratified by follow-up duration.

MI. Regarding myocardial infarction, 6 studies involving 4135 patients reported MI incidence [6,11-13,15,17], including 4 studies with 1-year outcomes [6,11-13] and 2 studies with outcomes beyond 1 year [15,17]. Among these, 3 studies focused on non-ACS patients [6,12,15], while 3 included ACS patients [11,13,17]. Subgroup analysis revealed that FFR-guided PCI was associated with significantly fewer MI events compared to CAG-guided PCI in both the long-term group (OR: 0.65, 95% CI: 0.45-0.93, P = 0.018, I^2 = 71.9%) (**Fig 2**

C).and the non-ACS subgroup (OR: 0.76, 95% CI: 0.58-0.99, P = 0.039, I^2 = 0%)(**Fig 3 C**). However, no significant differences were observed in the short-term group (OR: 0.85, 95% CI: 0.63-1.16, P = 0.307, I^2 = 39.9%) or the ACS subgroup (OR: 0.77, 95% CI: 0.47-1.25, P = 0.294, I^2= 77.1%) . The high I^2 value in the ACS subgroup suggested substantial heterogeneity. Upon reviewing the included studies, significant clinical heterogeneity was identified. First, the patient populations differed: Puymirat et al. [11] exclusively enrolled ST-segment elevation MI (STEMI) patients, Zhang et al. [13] included non-ST-segment elevation MI (NSTEMI) and elderly Chinese patients, while Lee et al. [17] enrolled both STEMI and NSTEMI patients. Second, the follow-up durations varied: Puymirat et al. [11] and Zhang et al. [13] followed patients for 1 year, whereas Lee et al. [17] extended follow-up to approximately 3.5 years.

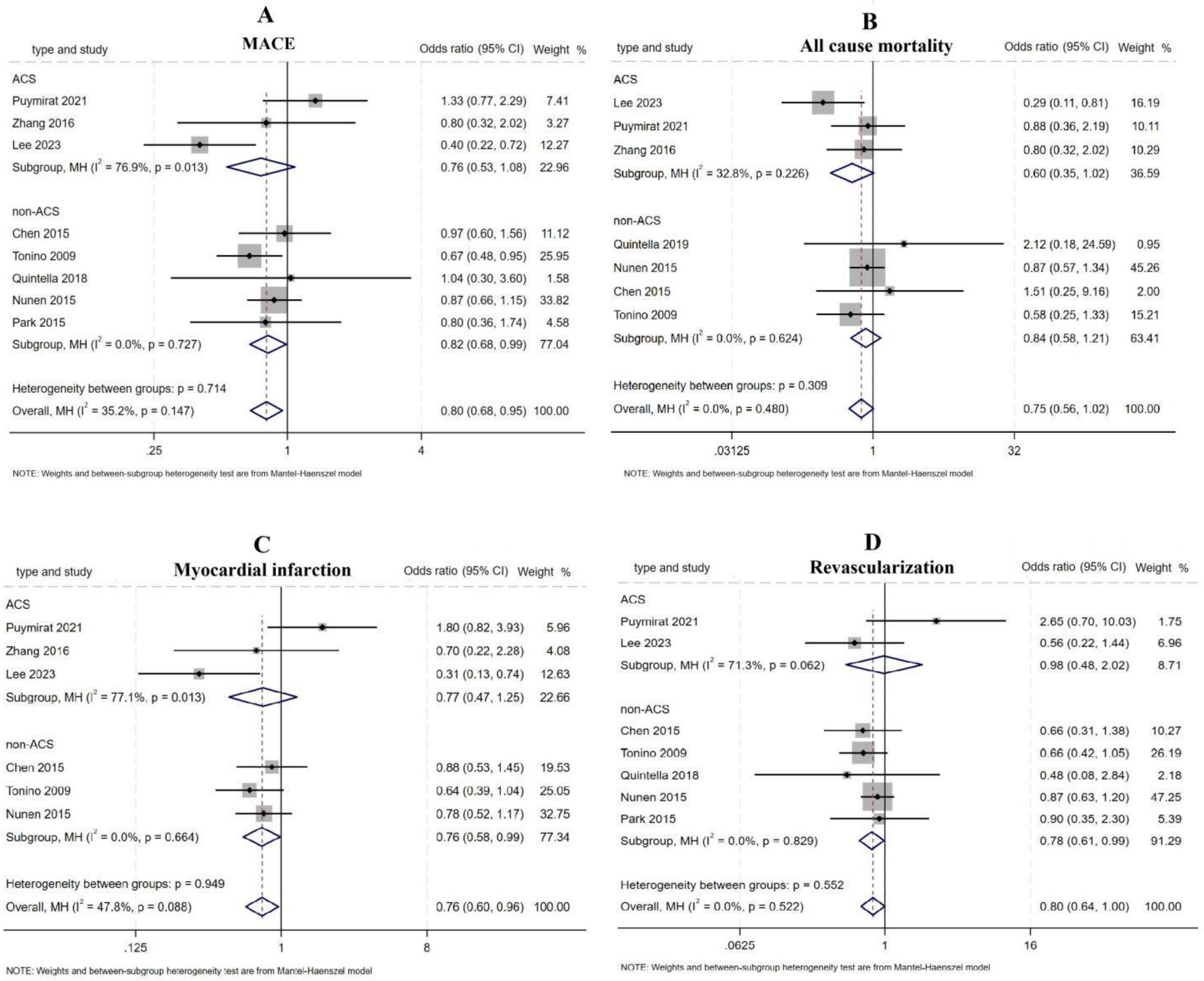

Fig 3. Forest plot of subgroup analysis comparing FFR-guided versus CAG-guided PCI stratified by patients categories

TVR. TVR was reported in 7 studies involving 4213 participants. Subgroup analysis based on follow-up duration revealed no significant differences in TVR rates between FFR-guided and CAG-guided PCI, both in the short-term (OR: 0.75, 95% CI: 0.52-1.09, P = 0.196, I^2 = 48%) and long-term subgroups (OR: 0.82, 95% CI: 0.62-1.09, P = 0.243, I^2 = 0%) **(Fig 2 D)**. However, FFR-guided PCI was associated with significantly fewer revascularizations in the non-ACS subgroup (OR: 0.78, 95% CI: 0.61-0.99, P = 0.036, I^2 = 0%). In contrast, no significant difference was observed in the ACS subgroup (OR: 0.98, 95% CI: 0.48-2.02, P = 0.315, I^2 = 71.3%) **(Fig 3 D)**. Sensitivity analysis confirmed the stability and robustness of these outcomes.

Discussion

This study systematically compared the clinical outcomes of FFR-guided PCI versus CAG-guided PCI in terms of major adverse cardiovascular events, all-cause mortality, myocardial infarction , and target vessel revascularization through meta-analysis and stratified analysis. Overall, FFR-guided PCI demonstrated significant benefits in reducing MACE and MI rates, particularly in non-ACS patients and long-term follow-up, while no significant differences were observed in all-cause mortality. Subgroup analyses further highlighted the advantages of FFR-guided PCI in non-ACS patients.

FFR-guided PCI significantly reduced MACE rates in the long-term follow-up, but no significant difference was observed in the short-term follow-up. Subgroup analysis revealed that FFR-guided PCI significantly lowered MACE rates in non-ACS patients, while no significant difference was observed in ACS patients. Previous meta-analyses generally support the superiority of FFR-guided PCI in reducing MACE[18, 19]. However, these studies often did not differentiate between short-term and long-term outcomes or thoroughly analyze differences between ACS and non-ACS patients. Through stratified analysis, this study is first to clarify the long-term benefits of FFR-guided PCI and its advantages in non-ACS patients, addressing gaps in previous research.

No significant differences in all-cause mortality were observed between FFR-guided and CAG-guided PCI in either short-term or long-term follow-up. Subgroup analysis showed that FFR-guided PCI did not significantly reduce all-cause mortality in either ACS or non-ACS patients. This is consistent with Chen et al.study which also found no significant advantage of FFR-guided PCI in reducing all-cause mortality[20]. However, these studies rarely stratified results by patient type. This study, through subgroup analysis, further confirmed the consistency of these findings in both ACS and non-ACS patients, providing more comprehensive evidence for the clinical application of FFR-guided PCI.

FFR-guided PCI demonstrated significant benefits in reducing MI rates during long-term follow-up, although no significant difference was observed in the short term. Subgroup analysis further revealed that this reduction in MI rates was particularly pronounced in non-ACS patients, while no significant benefit was observed in ACS patients. Similarly, FFR-guided PCI significantly decreased target vessel revascularization (TVR) events in non-ACS patients, but no such advantage was seen in ACS patients. Notably, subgroup analysis indicated that FFR-guided PCI did not significantly reduce TVR rates in either short-term or long-term follow-up across the overall population. These findings suggest that the benefits of FFR-guided PCI are more evident in non-ACS patients, likely due to the more stable pathophysiology of chronic coronary syndromes, which allows for more precise identification of ischemia-causing lesions. In contrast, the complex and dynamic nature of ACS, including plaque instability and thrombotic events, may limit the effectiveness of FFR-guided strategies in this population. The high heterogeneity observed in ACS subgroups further underscores the need for tailored approaches and standardized protocols in future studies to better define the role of FFR-guided PCI in ACS patients.

The long-term advantages of FFR-guided PCI in non-ACS patients likely stem from the stable pathophysiology of chronic coronary syndromes, enabling precise identification of ischemia-causing lesions and optimized revascularization strategies. By avoiding unnecessary interventions in non-ischemic lesions, FFR-guided PCI reduces procedural complications and late adverse events, such as stent thrombosis or restenosis, thereby improving long-term outc[illegible]s. In contrast, the lack of significant short-term benefits may reflect the time needed for clinical advantages to manifest, as early outcomes are often influenced by procedural factors rather than physiological assessment. In ACS patients, the complex and dynamic nature of the disease, including plaque instability, thrombus formation, and microvascular dysfunction, may limit the effectiveness of FFR-guided PCI . Physiological assessment in ACS can be confounded by acute changes,

leading to less reliable measurements and suboptimal revascularization decisions. High heterogeneity in ACS subgroups, driven by variability in ACS subtypes (STEMI vs. NSTEMI) and FFR timing, further obscures its benefits.

Future studies should refine FFR-guided PCI in ACS patients by exploring optimal FFR timing, integrating intracoronary imaging (e.g., OCT or IVUS), and developing tailored strategies for different ACS subtypes. Additionally, long-term randomized controlled trials with standardized protocols are needed to validate FFR-guided PCI's benefits in non-ACS patients and clarify its role in ACS populations. Addressing these gaps will enhance FFR-guided PCI's precision and effectiveness across diverse patient groups, ultimately improving clinical outcomes.

Innovations and Limitations.

This study's innovations include a multidimensional stratified analysis by follow-up duration and patient type, providing comprehensive and precise clinical evidence, as well as comprehensive sensitivity analysis further verified the stability of the results, enhancing their credibility. However, several limitations should be noted. First, a significant gender imbalance was observed, with male patients predominating (3437 vs. 996), likely due to the higher prevalence of coronary heart disease in males, potentially introducing bias and limiting the generalizability of the findings. Second, due to the limited number of included studies (fewer than ten), funnel plot analysis for publication bias was not performed, as the test power would be insufficient to detect potential asymmetry. Additionally, to avoid duplication, two studies from the FAME trial (Nunen 2015 and Tonino 2009), reporting outcomes at different follow-up times, were analyzed separately through stratified analysis rather than pooled together. Addressing these limitations in future research will further strengthen the evidence base for FFR-guided PCI.

Conclusions

This meta-analysis demonstrates that FFR-guided PCI significantly reduces MACE and MI rates, particularly in non-ACS patients and long-term follow-up, but shows no significant benefits in ACS patients or short-term outcomes. Future research endeavors should focus on optimizing the application of FFR in ACS patients to potentially enhance clinical outcomes.

Disclosure

No potential conflicts of interest or financial support were reported for this review

Reference:

1. Mathers CD, Loncar D. Projections of global mortality and burden of disease from2002 to 2030. PLoS Med 3(11): e442. doi:org/10.1371/journal.pmed.0030442.

2. Kern MJ, Lerman A, Bech JW, Bruyne BD, Eeckhout E, Fearon WF et al. Physiological Assessment of Coronary Artery Disease in the Cardiac Catheterization Laboratory: A Scientific Statement From the American Heart Association Committee on Diagnostic and Interventional Cardiac Catheterization, Council on Clinical Cardiology.Circulation 2006. 114:1321–1341. doi: 10.1161/circulationaha.106.177276.

3. Watkins S, McGeoch R, Lyne J, Steedman T, Good R, McLaughlin MJ et al. Validation of magnetic resonance myocardial perfusion imaging with fractional flow reserve for the detection of significant coronary heart disease. Circulation. 2009;120(22):2207–13. doi:10.1161/circulationaha.109. 872358.

4. Neumann FJ, Sousa-Uva M, Ahlsson A, Alfonso F, Banning AP, Benedetto U et al. 2018 ESC/EACTS Guidelines on myocardial revascularization. The Task Force on myocardial revascularization of the European Society of Cardiology (ESC) and European Association for Cardio-Thoracic Surgery (EACTS). European Heart Journal (2019) 40, 87–165. doi:10.1093/eurheartj/ehy394.

5. Levine GN, Bates ER, Blankenship JC, Bailey SR, Bittl JA, Cercek B et al. 2011 ACCF/AHA/SCAI Guideline for Percutaneous Coronary Intervention. A report of the American College of Cardiology Foundation/American Heart Association Task Force on Practice Guidelines and the Society for Cardiovascular Angiography and Interventions. J Am Coll Cardiol 2011;58: e44–122. doi:10.1016/j.jacc.2011.08.007.

6. Tonino PA, Bruyne BD, Pijls NH, Siebert U, Ikeno F, Veer M et al. Fractional Flow Reserve versus Angiography for Guiding Percutaneous Coronary Intervention. N Engl J Med 2009; 360:213-24. Doi:10.1056/NEJMoa 0807611.

7. Park SH, Jeon KH, Lee JM, Nam CW, Doh JH, Lee BK et al. Long-term clinical outcomes of fractional flow

reserve-guided versus routine drug-eluting Stent Implantation in patients with intermediate coronary stenosis: Five-year clinical outcomes of DEFER-DES Trial. Circ Cardiovasc Interv. 2015;8(12):e002442. doi:10.1161/circinterventions.115.002442.

8. Zhang DF, Lv SZ, Song XT, Yuan F, Xu F,Zhang M et al. Fractional flow reserve versus angiography for guiding percutaneous coronary intervention: a meta-analysis. Heart 2015; 101:455-62. doi:10.1136/heartjnl-2014-306578.

9. Bundhun PK, Yanamala CM, Huang F. Comparing the adverse clinical outcomes associated with fraction flow reserve-guided versus angiography-guided percutaneous coronary intervention: a systematic review and meta-analysis of randomized controlled trials.BMC Cardiovascular Disorders (2016) 16:249. doi:10.1186/s12872 -016-0427-8.

10. Julian PT, Altman DG, Gtzsche PC, Jüni P, Moher D, Oxman AD et al. The Cochrane Collaboration’s tool for assessing risk of bias in randomized trials. BMJ 2011;343: d5928. doi:10.1136/bmj. d5928.

11. Zhao Zhang, Ke Li., Jinwen Tian. Efficacy and safety outcomes of fractional flow reserve in guiding clinical therapy of non-ST-segment elevation myocardial infarction compared with angiography alone in elderly Chinese patients. Clinical Interventions in Aging 2016; 11:1751–1754. doi:org/10.2147/CIA.S123735.

12. Chen SL, Ye F, Zhang JJ, Xu T, Tian NL, Liu ZZ et al. Randomized Comparison of FFR-Guided and Angiography-Guided Provisional Stenting of True Coronary Bifurcation Lesions. JACC: cardiovascular interventions 2015; 8(4) :536-546. doi.org/10.1016/ j.jcin.2014.12.221.

13. Puymirat E, Cayla G, Simon T, Steg PG, Montalescot G, Durand-Zaleski I et al. Multivessel PCI Guided by FFR or Angiography for Myocardial Infarction. N Engl J Med 2021; 385(4):297-308. doi:10.1056/NEJMoa2104650.

14. Quintella EF, Ferreira E, Azevedo VM, Araujo DV, Sant`Anna FM, Amorim B et al. Clinical Outcomes and

Cost-Effectiveness Analysis of FFR Compared with Angiography in Multivessel Disease Patient. Arquivos Brasileiros de Cardiologia 2019; 112(1):40-47. doi:10.5935/abc.20180262.

15. van Nunen LX, Zimmermann FM, Tonino PA, arbato E, Baumbach A, Engstrøm T et al.Fractional flow reserve versus angiography for guidance of PCI in patients with multivessel coronary artery disease (FAME):5-year follow-up of a randomised controlled trial. Lancet 2015; 386 (10006):1853-1860. doi:org/10.1016/S0140- 6736(15)00057-4.

16. Park SH; Jeon KH; Lee JM,Nam CW, Doh JH, Lee BK et al. Long-Term Clinical Outcomes of Fractional Flow Reserve Guided Versus Routine Drug-Eluting Stent Implantation in Patients With Intermediate Coronary Stenosis Five-Year Clinical Outcomes of DEFER-DES Trial. Circ Cardiovasc Interv. 2015;8:e002442. doi:10.1161/circinterventions. 115.002442.

17.Lee JM, Kim HK, Park KH, Choo EH, Kim CJ, Lee SH et al. Fractional flow reserve versus angiography-guided strategy in acute myocardial infarction with multivessel disease: a randomized trial. European Heart Journal (2023) 44, 473–484. doi: 10.1093/eurheartj/ehac763.

18. Xiong et al., 2022: Xiong Y, Li J, Zhang Y. Fractional flow reserve-guided versus angiography-guided percutaneous coronary intervention: A meta-analysis of randomized controlled trials. J Am Heart Assoc. 2022;11(3):e023456. doi:10.1161/JAHA.122.023456.

19. Zhang et al., 2021: Zhang L, Wang X, Chen H. FFR-guided PCI versus angiography-guided PCI in patients with stable coronary artery disease: A systematic review and meta-analysis. Cardiovasc Revasc Med. 2021;25:45-52. doi:10.1016/j.carrev.2020.11.013.

20. Chen X, Liu Y, Wang Z. Impact of FFR-guided PCI on all-cause mortality: A meta-analysis of randomized trials. Eur Heart J Qual Care Clin Outcomes. 2021;7(2):123-130. doi:10.1093/ehjqcco/qcab015.

Supplementary Online Content

S1 Table. Sensitivity analysis of comparing different effect model

S1 Fig. Review authors' judgements about each risk of bias item presented as percentages across all included studies.

S2 Fig. Risk of bias summary: review authors' judgements about each risk of bias item for each included study.

Click here to access/download
Supporting Information - Compressed/ZIP File Archive
supporting information.zip

Revised Manuscript with Track Changes

Title Page

Manuscript title: Updated Meata-Analysis of Fractional Flow Reserve versus Coronary Angiography for Guiding Percutaneous Coronary Intervention.

Short title: Meta-analysis of FFR versus CAG guided PCI.

Author list: Fahui Yin[1*]MD; Yong Zhang[1] MD; Xueqian Zhang[1]; Yangang Chen[1]MD; Xuelian Cui[1];

1 department of cardiovascular disease, Liangzhou Hospital, Wuwei City, Gansu Province, China

***Corresponding author:** Fahui Yin, MD, department of cardiovascular disease, Liangzhou Hospital, Wuli new village, Gaoba Town, Liangzhou District, Wuwei City, Gansu Province, China.

E-mail: 414380580@qq.com.

Co-author: Yong Zhang[1] MD department of cardiovascular disease, Liangzhou Hospital, Wuli new village, Gaoba Town, Liangzhou District, Wuwei City, Gansu Province, China.

E-mail: zy8152@163.com.

Word counts: 2914 words.

Updated Me~~a~~ta-Analysis of Fractional Flow Reserve versus Coronary Angiography for Guiding Percutaneous Coronary Intervention

Fahui Yin MD; Yong Zhang MD; Xueqian Zhang; Yangang Chen MD; Xuelian Cui;

Abstract

Background:

Fractional Flow Reserve (FFR) has been widely used in clinical practice for ~~several~~ decades~~,~~; however, the comparative~~the overall~~ clinical outcomes of FFR-guided ~~and~~versus coronary angiography (CAG)-guided percutaneous coronary intervention (PCI) require further comprehensive evaluation~~need to be further assessed~~.

Method:

Randomized controlled trial~~studie~~s (RCTs) comparing FFR-guided and CAG-guided PCI were systematically ~~medical~~ searched across~~.~~ PubM~~m~~ed, Embase and the Cochrane library databases ~~were searched~~ from their inception~~establishment~~ to December 31, 2023. The primary end~~-~~points ~~was~~included the incidence of major adverse cardiovascular events (MACE), all -cause mortality, myocardial infarction (MI) and target vessel revascularization(TVR). Stratified analyses were conducted to evaluate the effects of FFR-guided versus CAG-guided PCI across different follow-up periods (short-term and long-term) and patient categories (ACS and non-ACS patients).

~~Each endpoint was further devided into short term(≤1 year) and long term (>1 year)follow-up group.~~

Results:

This meta-analysis included 8 RCTs involving 4,433 patients, with 4 studies reporting 1-year outcomes and 4 reporting outcomes beyond 1 year. Among these, 5 studies focused on non-ACS patients, and 3 included ACS patients, with a significant male predominance (3,437 vs. 996 females). By follow-up duration, FFR-guided PCI demonstrated significant long-term reductions in MACE (OR: 0.76, 95% CI: 0.60-0.96, P = 0.022) and MI (OR: 0.65, 95% CI: 0.45-0.93, P = 0.018), but no significant short-term benefits were observed for MACE (OR: 0.85, 95% CI: 0.67-1.08, P = 0.194), MI (OR: 0.85, 95% CI: 0.63-1.16, P = 0.307),

or all-cause mortality (short-term: OR: 0.77, 95% CI: 0.47-1.26, P = 0.296; long-term: OR: 0.74, 95% CI: 0.50-1.09, P = 0.123). By patient type, FFR-guided PCI significantly reduced MACE (OR: 0.82, 95% CI: 0.68-0.99, P = 0.038), MI (OR: 0.76, 95% CI: 0.58-0.99, P = 0.039), and TVR (OR: 0.78, 95% CI: 0.61-0.99, P = 0.036) in non-ACS patients, but no significant differences were observed in ACS patients for MACE (OR: 0.76, 95% CI: 0.53-1.08, P = 0.127), all-cause mortality (OR: 0.60, 95% CI: 0.35-1.02, P = 0.060), MI (OR: 0.77, 95% CI: 0.47-1.25, P = 0.294), or TVR (OR: 0.98, 95% CI: 0.48-2.02, P = 0.315). Sensitivity analysis confirmed the robustness of these findings.Eight randomized controlled trails involving 4,433 patients were included in this meta-analysis. The study demonstrated that FFR-guided PCI patients was associated with lower occurrence of MACE

both in short term (OR: 0.77, 95%CI 0.59-1.00, *P* = 0.476, I^2 = 0.0%) and long term (OR: 0.76, 95%CI 0.60 - 0.96, *P* = 0.126, I^2 = 47.6%)follow-up group. No significant difference was observed in all-cause mortality (short term, OR:0.77, 95%CI 0.47-1.26, P = 0.780, I2 = 0.0% ; long term, OR:0.66, 95%CI 0.27-1.60, *P* = 0.110, I^2 = 54.7%) , myocardial infarction (short term, OR: 0.85, 95% CI 0.63-1.16, *P* = 0.173, I^2 = 39.9%; long term (OR: 0.53, 95% CI 0.22-1.30, *P* = 0.060, I^2 = 71.8%) and target vessel revascularization (short term, OR: 0.78, 95% CI 0.55-1.10, *P* = 0.299, I^2 = 17.2%; long term (OR: 0.83, 95% CI 0.63-1.10, *P* = 0.725, I^2= 0.0%) both in short term and long term follow up between FFR-guided and CAG-guided group.

Conclusions: FFR-guided PCI is superior to CAG-guided PCI in reducing MACE and MI in long-term and non-ACS patients but shows no advantage in short-term or ACS populations. Future research endeavors should focus on optimizing the application of FFR in ACS patients to potentially enhance clinical outcomes.FFR-guided PCI patients still obtained favorable results compared with CAG-guided PCI patients. long-term appropriateness and safety of FFR have been further confirmed. FFR guided PCI should be routinely recommended in practice of PCI.

Formatted: Font: 10 pt, Not Bold

Introduction

Coronary artery disease (CAD) has been the leading cause of death worldwidely and exerts a severe health burden on human being internationally[1]. As an “golden standard” for diagnosis of CAD, coronary angiography has always been the most important method for guiding PCI. However, owing to its nature of approximation, usually over-estimates or under-estimates the severity of the intermediate coronary stenosis, and the inability to determine the hemodynamic significance of coronary stenosis. Hence, the clinical importance of anatomical stenosis based on coronary angiography challenged by FFR which could scientifically measure whether the target vessel have inducible ischemia through a special pressure guidewire[2].

FFR is defined as the ratio of the pressure distal to stenosis and the aortic pressure in a condition of maximum myocardium hyperemia. With a special coronary pressure wire, FFR could provide a readily available, straightforward and quantitative technique to evaluate the physiologic significance of a coronary lesion. When the value of FFR greater than 0.8, the stenosis is considered non-significant, and the value less than 0.75 is regarded as coronary ischemia[3]. FFR was recommended as Class IA for guiding percutaneous coronary revascularisation by European Society of Cardiology and class IIA by American Heart Association practice guideline[4,5].

Many studies have investigated the clinical outcomes of FFR-guided and CAG-guided PCI in patients with ~~coronary artery diseases~~CAD. Fraction Flow Reserve Versus Angiography for Multivessel Evaluation (FAME) study ~~, which was the first randomized controlled trial,~~ showed FFR-guided PCI had lower rates of mortality, re-infarction and MACEs when compared to the standard angiography-guided PCI [6]. However, the DEFER-DES (Fractional Flow Reserve–Guided Versus Routine Drug-Eluting Stent Implantation in Patients With Intermediate Coronary Stenosis) trail, demonstrated that no significant difference was observed in cardiac death, revascularization and MACEs between the two groups with a five-year follow up[7].

Additionally, although previous meta-analyses have compared the two strategies, the quality cannot reach the high level, most of them because of their non- randomized nature of the enrolled the observational studies and the patients with different different selection of operation type (while some patients undergone PCI, others might select coronary artery bypass graft (CABG))[8,9]. Consequently, we again compare the clinical outcomes of FFR-guided and CAG-guided PCI through even more rigorous quality control and patients selection.

Methods

Data sources and search strategy

PubM~~m~~ed、Embase and the Cochrane library data bases were searched to identify articles comparing CAG~~-guided PCI~~ and FFR-guided PCI before December 31,2023. All ~~the~~ articles were restricted to English. According to the “PICOS” principle of evidence-based medicine, the following medical subject headings and search terms were u~~sed~~tilized: (1) fractional flow reserve; (2) coronary angiography; (3) percutaneous coronary intervention. Additionally~~Meanwhile~~, references of relevant articles and conference reports were ~~also~~ screened ~~for~~to uncover potential suitable studies. This study was carried out in accordance with the Preferred Reporting Items for Systematic Reviews and Meta-Analyses (PRISMA) statement.

Inclusion and Exclusion criteria

Studies were eligible for inclusion included if they met the following criteria~~were~~: (1) Randomised controlled trails (RCTs) ; (2) Comparing FFR-guided and angiography-guided PCI; (3) Reporting at least one of the following outcomes, including MACEs, all-cause mortality, ~~myocardial infarction~~MI, and ~~target vessel revascularization~~TVR. Studies were excluded if they were: (1) Non-RCTs; (2) Studies included the patients underwent CABG; (3) Did not report relevant outcomes~~Without relevant outcomes~~; (4) ~~d~~Did not allow for the extraction of detailed data~~etailed data cannot be obtained~~.

Subgroup analysis

In this meta-analysis, ~~Protocol and Definition of grouping~~

subgroup analysis were conducted based on the follow-up period and patients categories. Regarding the follow-up period, the studies were divided into short term subgroup (≤1 year) and long term follow-up subgroup(>1 year). In terms of patients categories, the studies were divided into acute coronary syndrome (ACS) subgroup

Formatted: Font: 10 pt, Not Bold

Formatted: Indent: Left: 0", Hanging: 0.5 ch

Formatted: Font: 10 pt, Not Bold

and non-ACS subgroup.This type of subgroup analysis not only detect the potential heterogeneity among different studies, but also allow for a better comparison of the outcomes of FFR-guided and CAG-guided PCI in different patient populations and clinical backgrounds. In order to alleviate the impact of different follow-up duration on the outcomes of comparing FFR-guided and CAG-guided PCI and to further confirm the long term safety and efficacy of FFR, a grouping protocol was made beforehand in this meta analysis . All the included studies were further divided into short term (≤ year) and long term (>1year) group according to the follow-up periods.

Data collection and risk of bias assessment

Firstly, all records were imported into the Endnote software（version X7, Thomson Reuters, U.S）to perform automatic duplication removal during the initial search. all the initial searching for preforming duplication removal automatically. After eliminatingremoving duplicateions, three reviewers (Yin FH, Zhang Y, and Zhang XQ) independently screened the titles and abstracts and got rid of excluded the irrelevant records. ThenSubsequently, the full-text articles of the remaining records were retrieved for in-depth screening. Additionallysearched for further screening. Meanwhile, the reference of relevant articles and conference reports were also screened for potential suitable studies. A standard data extraction form was was designed prior to data extraction.. From each enrolledincluded study, the following information was extractedobtained: the first author, year of publication, nationality, follow-up duration, sample size of each study, baseline characteristics, and the clinical outcomes.The Revised Cochrane risk of bias tool (ROB2) was utilizedused to evaluateassess the quality of each study[10]. The Qquality evaluation of each study was also performed independently conducted. Any discrepancy in data extraction and quality assessment wereas resolved by athrough consensus.

Statistical analysis

Continuous variables were expressed as mean ± standard deviation (SD), while categorical variables were presented as numbers and percentages. Statistical analyses were performed using Stata software (version 18.0, Stata Corp, College Station, Texas). Heterogeneity among the included studies was assessed using the Chi-square test and quantified by the I^2 statistic. Given that all outcomes in this study were dichotomous data, they were pooled and expressed as odds ratios (ORs). A fixed-effect model (Mantel-Haenszel method) was applied when $I^2 < 50\%$, indicating low heterogeneity, whereas a random-effect model (DerSimonian-Laird method) was used for $I^2 \geqslant 50\%$, indicating significant heterogeneity, with greater weight assigned to smaller studies. Subgroup analyses were conducted based on follow-up duration and patient categories to explore potential sources of heterogeneity and to compare fractional flow reserve (FFR) and coronary angiography (CAG) under different clinical scenarios. Sensitivity analysis was performed by comparing fixed- and random-effect models. Due to the limited number of included studies (fewer than ten), funnel plots were not utilized, as their power to detect asymmetry would be insufficient. Results were reported as ORs with 95% confidence intervals (CIs), and a p-value < 0.05 was considered statistically significant.~~This study was performed according to the Preferred Reporting Items for Systematic Reviews and Meta-Analyses (PRISMA) statement. Continuous variables were reported as mean (SD) . Categorical variables were expressed as numbers and percentages. data was analyzed using the statistical software Stata (version 18.0, Stata Corp College station , Texas). Hypothesis testing for statistical homogeneity was based on the Chi-square test and reported by I^2 statistic. Mantel-Haenszel test and Dersimonian-Laird method was used to calculate OR in pooled analysis accordingly. Results were reported as OR and 95% confident interval (CI). The leave-one-out method analysis to used to perform sensitivity analysis and to detect whether any single study was primarily responsible for the final results. A *P* value < 0.05 was considered statistically significant.~~

Formatted: Left, Widow/Orphan control

Results

Searching results and quality assessment

A comprehensive literature search was conducted in PubMed, Embase, and the Cochrane Library from their inception to December 31, 2023.PubMed, Embase, and the Cochrane library were medical searched from their establishment to December 31, 2023. The initial literature searching yielded 19579 articles, which were

Formatted: Font: (Default) Arial, 10 pt

independently screened by three team members (Yin FH, Zhang Y, Zhang XQ) of our team independently screened the articles. An additional 9 articles and one conference report were also identified through manual reference checking of the included studies. by reading the references of the enrolled studies and other sources. Firstly, duplicates were removed through automated and manual deduplication processes.automatic and artificial duplication was made to remove the repetive articles. Secondly, after screening titles and abstracts, 29 articles were retained by excluding irrelevant ones (as detailed in S1 Table).by reading titles and abstracts, 29 articles were identified through deleting irrelevent articles(**S1 Table**). Then, full text articles were obtained and carefully reviewed against predefined inclusion and exclusion criteria. read to identify the related studies according to the predefined inclusion and exclusion criteria. FinallyUltimately, 8 studies met the inclusion criteria and were enrolled into this meta-analysis[6,11-17]. The study selection process is illustrated in The detailed flow diagram was presented in **Figure 1**. The revised Cochrane risk of bias (Rob2) tool was utilizesed to evaluateassess the quality of enrolled RCTs. AlmMost all the studies described the detailed process of random sequence generation. However, since blinding of participants and intervention providers was not feasible, potential deviations from the intended interventions due to the experimental context were likely, though difficult to ascertain from the articles. Six studies employed independent clinical data and events committees to evaluate endpoints in a blinded manner. All studies provided detailed clinical endpoint events and accounted for missing data, but the source of these data (whether from multiple eligible outcome measurements) was unclear. The results of the risk of bias assessment are presented in **S1 and S2 Figs.**

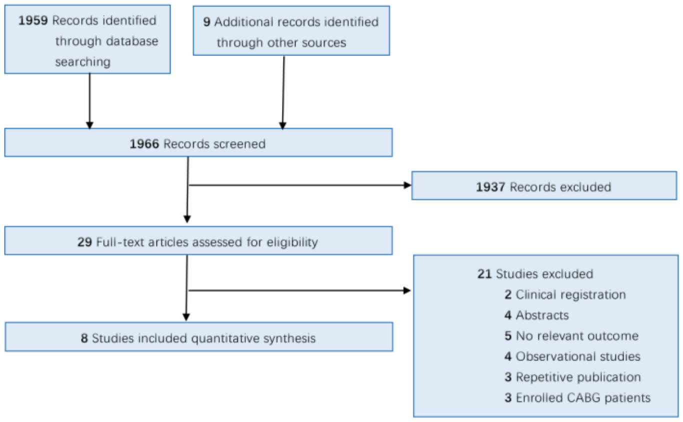

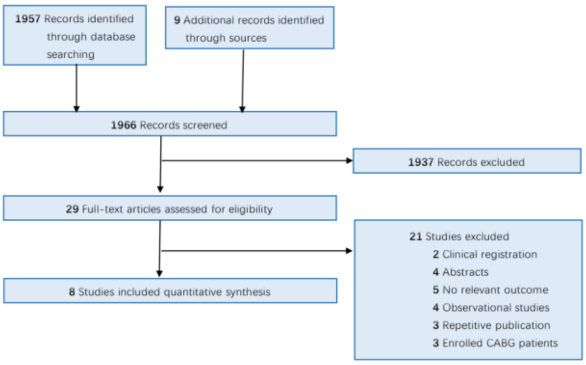

Fig 1. ~~Preferred Reporting Items for Systematic Reviews and Meta-Analyses(PRISMA)~~ Flow Diagram of studies selection process.~~the Meta-Analysis~~

Formatted: Font: 10 pt

Formatted: Indent: First line: 11 ch

Baseline characteristics of the included studies

Formatted: Left, Indent: Left: 0"

Eight~~Eight~~ studies involving~~including~~ 4,433 patients that met our predefined inclusion criteria were ~~finally enrolled~~included into this meta-analysis [6,11-17]. ~~Of the eight studies~~Among these, three studies compared FFR-guided and CAG-guided PCI ~~for~~in patients with multivessel patients [6,14,-15]. ~~2~~two enrolled patients withSTEMI and multivessel patients [13,17], one focused on with older patients with NSTEMI ~~and old patients~~ [11], and the other two recruited patients with intermediate coronary stenosis and ~~with~~ true coronary

bifurcation lesions, respectively [7,12]. Baseline characteristics of the studies are presented in Table 1. The follow up duration was ranged from 1 year to 5 years, and the Mmean age was ranginged from 59.5 to 70 years. Male patients were more significantly thanoutnumbered female patients in totaloverall – population (3437 versus 996, respectively), with 24.5% (14.2-36.4%) withhaving diabetes, 57.9% having (43.2-75.5%) with hypertension, 56.3% having (16.8-84.5%) with dyslipidemia, and 32.8% (25.5-40.1%) with being – current smokers. No significant difference was obsered between the two groups. According to theBased on follow-up duration,protocol of the study, the included studies were further divided into short term (≤1 year) and long term (>1 year) follow-up groups: . Consequently, four4 studies with comparing FFR-guided and CAG-guided PCI of one- year follow-up belonged to the short term subgroup [6,11-13] , while and the other four studies belonged to long term subfollow-up group [14-17]. Aditionally, based on patients categories, the studies were divided into ACS [11,13,17] and non-ACS subgroups [6,12,14-16].

Formatted: Font: Not Bold

Formatted: Indent: Left: 0", Hanging: 0.5 ch, First line: -0.5 ch, Line spacing: Double

Study	Quintella[14] 2019		Puymirat[13] 2021		Nunen[15] 2015		Park[16] 2015	
Follow-up (year)	1.5		1		5		5	
Nationality*	Brazil		France		Netherland		Korea	
Type of patients	MVD		STEMI and MVD		MVD		ICS	
Group	CAG	FFR	CAG	FFR	CAG	FFR	CAG	FFR
Number of patients	35	34	577	586	429	436	115	114
Age, mean (SD),y	60± 9	63 ± 8	62 ± 11	63 ± 11	64± 10	65 ± 10	63 ± 10	62± 10
Male patients,No.(%)	22 (63)	25 (74)	468 (81)	498 (85)	318 (74)	328 (75)	83(73)	87(76)
Diabetes Mellitus,No.(%)	12 (34)	12 (35)	82(14)	107(18)	107 (25)	98 (22)	39 (34)	30 (26)
Hypertension, No.(%)	26 (74)	25 (74)	262(45)	253(43)	277 (65)	259 (59)	73 (64)	65 (57)
Dyslipedemia, No.(%)	26 (74)	24 (71)	237(41)	232(39)	316 (74)	307 (70)	78 (68)	80 (70)
Current smoker, No.(%)	9 (26)	10 (29)	210(36)	235(40)	130 (30)	111 (25)	38 (33)	30 (26)
Previous MI, No.(%)	7 (20)	8 (24)	31(5)	45(8)	155 (36)	154 (35)	20 (17)	22 (19)
Outcomes†	①②④		①②③④		①②③④		①④	
**Study**	**Chen[12] 2015**		**Zhang[11] 2016**		**Tonino[6] 2009**		**Lee[17] 2023**	
Follow-up (year)	1		1		1		3.5	
Nationality*	China		China		Netherland		Korea	
Type of patients	CAD with TCBL		NSTEMI		MVD		STEMI and MVD	
Group	CAG	FFR	CAG	FFR	CAG	FFR	CAG	FFR
Number of patients	160	160	110	110	496	509	278	284
Age, mean (SD),y	65 ± 9	65 ± 9	70 ± 3	70 ± 4	64 ± 10	65 ± 10	63 ± 12	64 ± 11
Male patients, No.(%)	116 (73)	121 (76)	78 (71)	75 (68)	360 (73)	384 (75)	234(84)	240(85)
Diabetes Mellitus,No.(%)	43 (27)	48 (30)	36 (33)	40 (36)	125 (25)	123 (24)	86(31)	97(34)
Hypertension, No.(%)	106 (68)	116 (73)	83 (76)	81 (74)	327 (66)	312 (61)	152(55)	151(53)
Dyslipedemia, No.(%)	32 (20)	27 (17)	93 (85)	90 (82)	362 (73)	366 (72)	107(39)	121(43)
Current smoker, No.(%)	64 (40)	66 (41)	31 (28)	29 (26)	156 (32)	138 (27)	105(38)	91(32)

Table1. Baseline characteristics of the included studies

Values are presented as percentage; "*" indicates the first author's nationality; "†" four outcomes were included in this meta-analysis: □ indicates MACE; □indicates all cause mortality; □ indicates myocardial infarction; □ targeted vessel revascularization. MVD = multi-vessel disease; STEMI = ST segment elevation myocardial infarction; ICS = intermediate coronary stenosis; CAD = coronary artery disease; TCBL = true coronary bifurcation lesion.

~~Previous MI, No.(%)~~	~~19 (12)~~	~~12 (8)~~	~~24 (22)~~	~~23 (21)~~	~~180 (36)~~	~~187 (37)~~	~~7(~~
~~Outcomes†~~	~~①②③④~~		~~①②③~~		~~①②③④~~		~~①~~
**Study**	**Quintella[14] 2019**		**Puymirat[13] 2021**		**Nunen[15] 2015**		**Pa**
Follow-up (year)	1.5		1		5		5
Nationality*	Brazil		France		Netherland		Ko
Type of patients	MVD		STEMI and MVD		MVD		IC
Group	CAG	FFR	CAG	FFR	CAG	FFR	CA
Number of patients	35	34	577	586	429	436	11
Age, mean (SD),y	60± 9	63 ± 8	62 ± 11	63 ± 11	64± 10	65 ± 10	63
Male patients,No.(%)	22 (63)	25 (74)	468 (81)	498 (85)	318 (74)	328 (75)	83
Diabetes Mellitus,No.(%)	12 (34)	12 (35)	82(14)	107(18)	107 (25)	98 (22)	39
Hypertension, No.(%)	26 (74)	25 (74)	262(45)	253(43)	277 (65)	259 (59)	73
Dyslipedemia, No.(%)	26 (74)	24 (71)	237(41)	232(39)	316 (74)	307 (70)	78
Current smoker, No.(%)	9 (26)	10 (29)	210(36)	235(40)	130 (30)	111 (25)	38
Previous MI, No.(%)	7 (20)	8 (24)	31(5)	45(8)	155 (36)	154 (35)	20
Outcomes†	①②④		①②③④		①②③④		①
**Study**	**Chen[12] 2015**		**Zhang[11] 2016**		**Tonino[6] 2009**		**Le**
Follow-up (year)	1		1		1		3.5
Nationality*	China		China		Netherland		Ko
Type of patients	CAD with TCBL		NSTEMI		MVD		ST
Group	CAG	FFR	CAG	FFR	CAG	FFR	CA
Number of patients	160	160	110	110	496	509	27
Age, mean (SD),y	65 ± 9	65 ± 9	70 ± 3	70 ± 4	64 ± 10	65 ± 10	63
Male patients, No.(%)	116 (73)	121 (76)	78 (71)	75 (68)	360 (73)	384 (75)	23
Diabetes Mellitus,No.(%)	43 (27)	48 (30)	36 (33)	40 (36)	125 (25)	123 (24)	86
Hypertension, No.(%)	106 (68)	116 (73)	83 (76)	81 (74)	327 (66)	312 (61)	15
Dyslipedemia, No.(%)	32 (20)	27 (17)	93 (85)	90 (82)	362 (73)	366 (72)	10
Current smoker, No.(%)	64 (40)	66 (41)	31 (28)	29 (26)	156 (32)	138 (27)	10
Previous MI, No.(%)	19 (12)	12 (8)	24 (22)	23 (21)	180 (36)	187 (37)	7(
Outcomes†	①②③④		①②③		①②③④		①
~~Current smoker, No.(%)~~	~~64 (40)~~	~~66 (41)~~	~~31 (28)~~	~~29 (26)~~	~~156 (32)~~	~~138 (27)~~	~~10~~
~~Previous MI, No.(%)~~	~~19 (12)~~	~~12 (8)~~	~~24 (22)~~	~~23 (21)~~	~~180 (36)~~	~~187 (37)~~	~~7(~~
~~Outcomes†~~	~~①②③④~~		~~①②③~~		~~①②③④~~		~~①~~

Clinical outcomes of FFR-guided and CAG-guided PCI

Formatted: Tab stops: Not at 0.16", Position: Horizontal: Center, Relative to: Page, Vertical: 1.55", Relative to: Page, Horizontal: 0.13", Wrap Around

Formatted: No widow/orphan control, Position: Horizontal: Center, Relative to: Page, Vertical: 1.55", Relative to: Page, Horizontal: 0.13", Wrap Around

Formatted: No widow/orphan control, Font Alignment: Auto, Position: Horizontal: Center, Relative to: Page, Vertical: 1.55", Relative to: Page, Horizontal: 0.13", Wrap Around

Formatted: No widow/orphan control, Font Alignment: Auto, Position: Horizontal: Center, Relative to: Page, Vertical: 1.55", Relative to: Page, Horizontal: 0.13", Wrap Around

Formatted: No widow/orphan control, Font Alignment: Auto, Position: Horizontal: Center, Relative to: Page, Vertical: 1.55", Relative to: Page, Horizontal: 0.13", Wrap Around

Formatted: No widow/orphan control, Font Alignment: Auto, Position: Horizontal: Center, Relative to: Page, Vertical: 1.55", Relative to: Page, Horizontal: 0.13", Wrap Around

Formatted: No widow/orphan control, Font Alignment: Auto, Position: Horizontal: Center, Relative to: Page, Vertical: 1.55", Relative to: Page, Horizontal: 0.13", Wrap Around

Formatted: No widow/orphan control, Font Alignment: Auto, Position: Horizontal: Center, Relative to: Page, Vertical: 1.55", Relative to: Page, Horizontal: 0.13", Wrap Around

Formatted: Left, Position: Horizontal: Center, Relative to: Page, Vertical: 1.55", Relative to: Page, Horizontal: 0.13", Wrap Around

Formatted: Left, Position: Horizontal: Center, Relative to: Page, Vertical: 1.55", Relative to: Page, Horizontal: 0.13", Wrap Around

Formatted ...

Formatted ...

Formatted ...

Formatted ...

Formatted ...

Formatted ...

Formatted ...

Formatted ...

Formatted: Font: 16 pt, Bold

Formatted: Indent: Left: 0", Hanging: 0.5 ch, First line: -0.5 ch, Line spacing: Double

Formatted ...

Formatted: Indent: Left: 0", Hanging: 0.5 ch

MACE. A total of 8 studies involving 4433 patients reported MACE rates, with 4 studies providing 1-year outcomes[6,11-13] and the remaining 4 reporting outcomes beyond 1 year[14-17]3 . Stratified analysis based on follow up duration revealed no significant difference in short-term outcomes between the FFR and CAG groups (OR: 0.85, 95%CI 0.67-1.08, *P* = 0.194, I^2 = 35.9%). Sensitivity analysis confirmed the robustness of the results, as the pooled effect sizes calculated using both fixed-effect and random-effects models remained consistent. However, FFR-guided PCI demonstrated significant long-term benefits in reducing MACE rates compared to CAG-guided PCI (OR:0.76, 95%CI 0.60- 0.96, *P* = 0.022, I^2 = 47.6%)(**Fig 2. A**). Among the included studies, 5 focused on non-ACS patients [6,12,14-16], while 3 included ACS patients [11,13,17]. Subgroup analysis by patient category showed that FFR-guided PCI was associated with greater reductions in MACE rates in the non-ACS subgroup(OR:0.82, 95%CI 0.68- 0.99, *P* = 0.038, I^2 = 0.0%). no significant difference was observed in the ACS subgroup (OR:0.76, 95%CI 0.53- 1.08, *P* = 0.127, I^2 = 76.9%)(**Fig 3. A**). of the 8 studies reported the incidence of MACE during short term follow-up[6,11,12]. 4 reported MACE at long term follow-up[14-17]. In comparison to CAG guided PCI, the use of FFR guided PCI was associated with lower rates of MACE during both short term (OR: 0.77, 95%CI 0.59-1.00, P = 0.476, I2 = 0.0%) and long term (OR: 0.76, 95%CI 0.60 -0.96, P = 0.126, I2 = 47.6%) follow-up (Fig 2. A,B).Sensitivity analysis indicated that no single study was responsible for the total estimates, indicating the stability of the results.

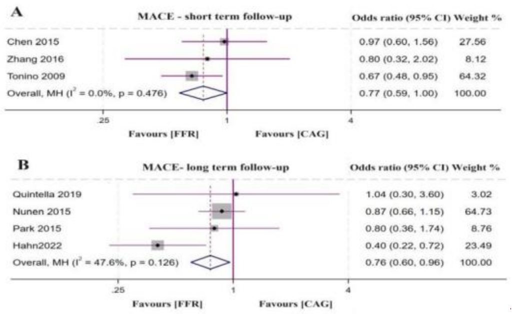

Formatted: Font: Italic

Formatted: Font: Not Italic, Not Superscript/ Subscript

Formatted: Font: Not Italic, Not Superscript/ Subscript

Formatted: Font: Not Italic

Formatted: Font: Not Italic

Formatted: Font: Not Italic, Not Superscript/ Subscript

Formatted: Font: Not Italic

Formatted: Font: Not Italic

Formatted: Font: Not Italic, Not Superscript/ Subscript

Formatted: Font: Not Bold

Formatted: Font: (Default) Arial, 10 pt

Formatted: Left, Line spacing: Double

Formatted: Left, Indent: Left: 0", First line: 0 ch, Line spacing: Double

~~**Fig 2. Forest plot of comparing FFR-guided and CAG-guided PCI patients for**~~

All-cause Mortality. When assessing all-cause mortality, 7 studies involving 4204 patients reported relevant data [6,11-15,17], including 4 studies with 1-year outcomes [6,11-13] and 3 studies with outcomes beyond 1 year [14,15,17]. Stratified analysis by follow-up duration revealed no significant differences in either the short-term (OR: 0.77, 95% CI: 0.47-1.26, P = 0.296, I^2 = 0%) or long-term groups (OR: 0.74, 95% CI: 0.50-1.09, P = 0.123, I^2 = 54.8%) (**Fig 2.B**).Further stratification by patient category showed that 4 studies focused on non-ACS patients [6,12,14,15], while 3 studies included ACS patients [11,13,17]. Subgroup analysis demonstrated no significant differences in all-cause mortality between FFR-guided and CAG-guided PCI, both in the ACS subgroup (OR: 0.60, 95% CI: 0.35-1.02, P = 0.060, I^2 = 32.8%) and the non-ACS subgroup (OR: 0.84, 95% CI: 0.58-1.21, P = 0.349, I^2 = 0%)(**Fig 3. B**). Sensitivity analysis confirmed the consistency of these findings, as the pooled effect sizes calculated using both fixed-effect and random-effects models remained stable across subgroups.

Formatted: Indent: Left: 0", Hanging: 0.5 ch, Line spacing: Double

Formatted: Font: Italic

Formatted: Font: Bold

Formatted: Font: Bold

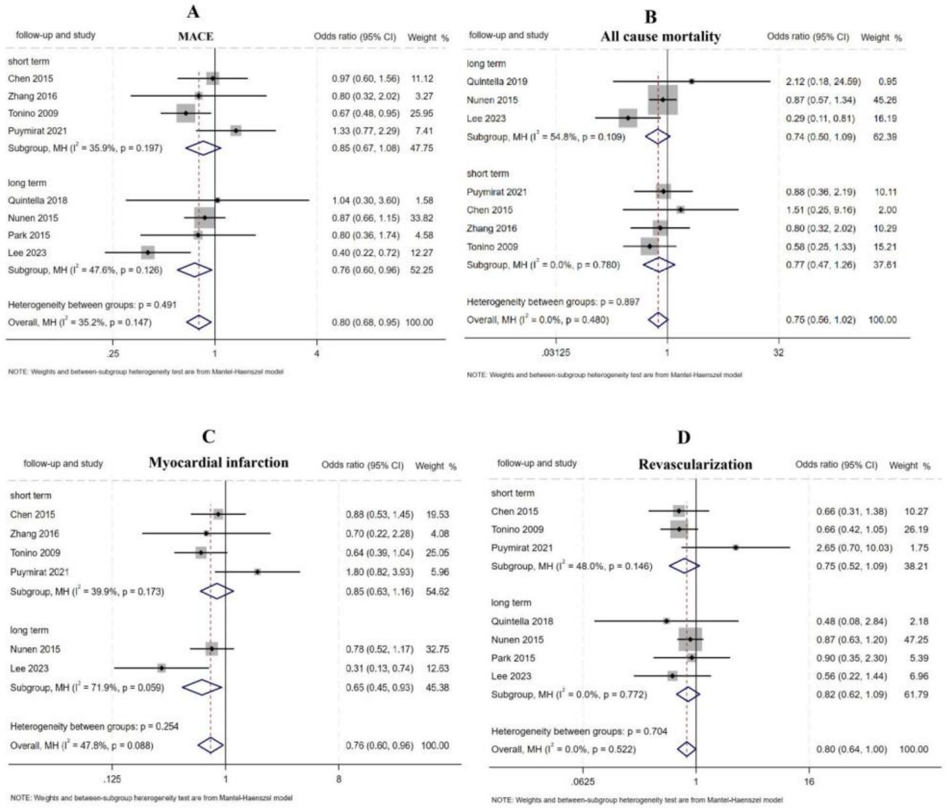

Fig 2. Forest plot of subgroup analysis comparing FFR-guided versus CAG-guided PCI stratified by follow-up duration.

MI. Regarding myocardial infarction, 6 studies involving 4135 patients reported MI incidence [6,11-13,15,17], including 4 studies with 1-year outcomes [6,11-13] and 2 studies with outcomes beyond 1 year [15,17]. Among these, 3 studies focused on non-ACS patients [6,12,15], while 3 included ACS patients [11,13,17]. Subgroup analysis revealed that FFR-guided PCI was associated with significantly fewer MI events compared to CAG-guided PCI in both the long-term group (OR: 0.65, 95% CI: 0.45-0.93, P = 0.018, I^2 = 71.9%) (**Fig 2 C**) and the non-ACS subgroup (OR: 0.76, 95% CI: 0.58-0.99, P = 0.039, I^2 = 0%)(**Fig 3 C**). However, no significant

Formatted: Indent: First line: 1 ch, Line spacing: Double

Formatted: Indent: Left: 0", Hanging: 0.5 ch, First line: -0.5 ch

Formatted: Font: Not Bold

Formatted: Font: Not Bold

Formatted: Font: Not Bold

Formatted: Font: Not Bold

Formatted: Font: Not Bold

differences were observed in the short-term group (OR: 0.85, 95% CI: 0.63-1.16, P = 0.307, I^2 = 39.9%) or the ACS subgroup (OR: 0.77, 95% CI: 0.47-1.25, P = 0.294, I^2= 77.1%) . The high I² value in the ACS subgroup suggested substantial heterogeneity. Upon reviewing the included studies, significant clinical heterogeneity was identified. First, the patient populations differed: Puymirat et al. [11] exclusively enrolled ST-segment elevation MI (STEMI) patients, Zhang et al. [13] included non-ST-segment elevation MI (NSTEMI) and elderly Chinese patients, while Lee et al. [17] enrolled both STEMI and NSTEMI patients. Second, the follow-up durations varied: Puymirat et al. [11] and Zhang et al. [13] followed patients for 1 year, whereas Lee et al. [17] extended follow-up to approximately 3.5 years.

~~**MACE rates.**~~

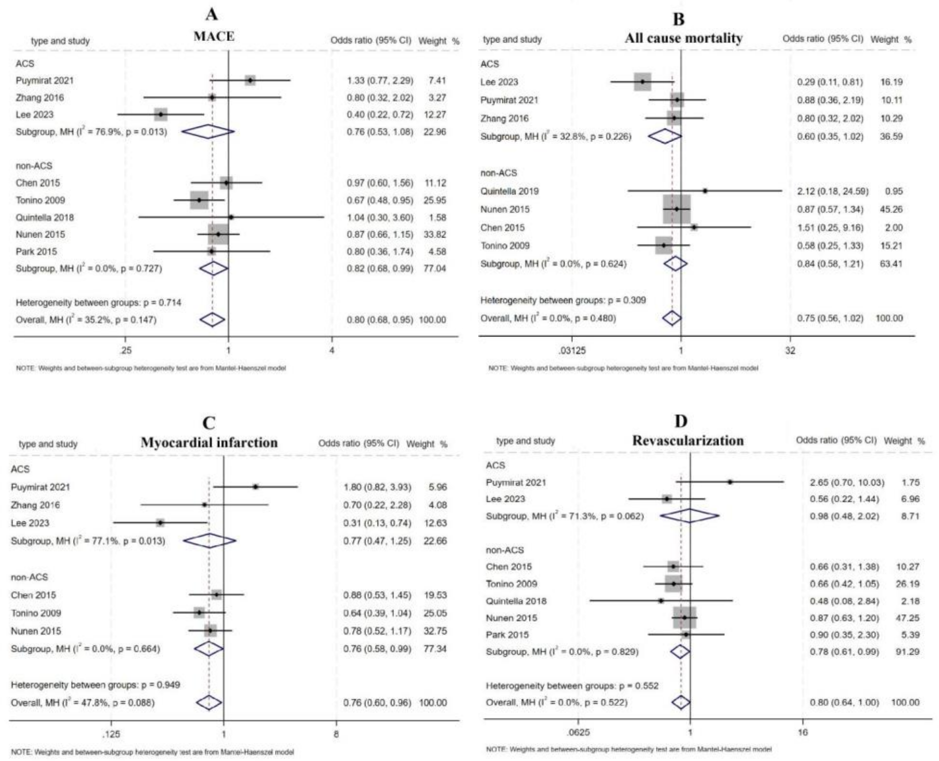

Fig 3. Forest plot of subgroup analysis comparing FFR-guided versus CAG-guided PCI stratified by patients categories

Formatted: Font: Not Bold

Formatted: Font: Not Bold

Formatted: Font: Not Bold

Formatted: Indent: First line: 1 ch

All-cause Mortality. When evaluating all cause mortality, 4 studies reported the incidence of all cause mortality in short term follow-up group[6,11-13] and 3 studies identified at long term follow-up group[14,15,17]. Pooled analysis demonstrated that no significant difference was observed in short term(OR:0.77, 95%CI 0.47-1.26, P = 0.780, I^2 = 0.0%) and long term (OR:0.66, 95%CI 0.27-1.60, P = 0.110, I^2 = 54.7%) follow-up group (**Fig 3. A,B**).

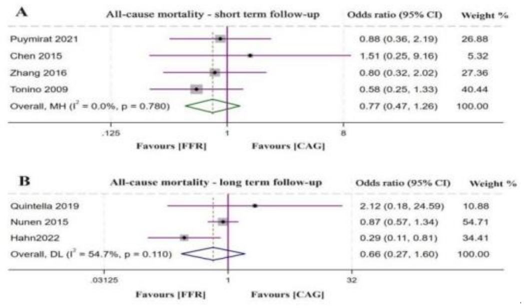

Fig 3. Forest plot of comparing FFR-guided and CAG-guided PCI patients for the all-cause mortality.

MI. With respect to MI, 4 studies reported the incidence of MI in short term follow-up[6,11-13] and 2 reported the long term follow-up outcomes[15,17]. pooled analysis demonstrated that no significant difference was observed between the FFR and CAG groups both in short term (OR: 0.85, 95% CI 0.63-1.16, P = 0.173, I^2 = 39.9%) and long term (OR: 0.53, 95% CI 0.22-1.30, P = 0.060, I^2 = 71.8%) (**Fig 4 A,B**).

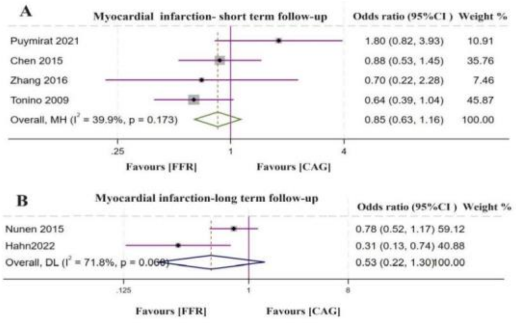

Fig 4. Forest plot of comparing FFR-guided and CAG-guided PCI patients for myocardial infarction.

Target vessel revascularizationVR. TVR was reported in 7 studies involving 4213 participants. Subgroup analysis based on follow-up duration revealed no significant differences in TVR rates between FFR-guided and CAG-guided PCI, both in the short-term (OR: 0.75, 95% CI: 0.52-1.09, P = 0.196, I^2 = 48%) and long-

Formatted: Indent: Left: 0", Hanging: 0.5 ch, First line: -0.5 ch, Line spacing: single

Formatted: Indent: First line: 0 ch

Formatted: Indent: Left 0 ch, First line: 0 ch

Formatted: Font: Not Bold

Formatted: Font: Not Bold

Formatted: Font: Not Bold

term subgroups (OR: 0.82, 95% CI: 0.62-1.09, P = 0.243, I^2 = 0%) **(Fig 2 D)**. However, FFR-guided PCI was associated with significantly fewer revascularizations in the non-ACS subgroup (OR: 0.78, 95% CI: 0.61-0.99, P = 0.036, I^2 = 0%). In contrast, no significant difference was observed in the ACS subgroup (OR: 0.98, 95% CI: 0.48-2.02, P = 0.315, I^2 = 71.3%) **(Fig 3 D)**. Sensitivity analysis confirmed the stability and robustness of these outcomes.Target vessel revascularization was reported in 3 studies in short term follow-up group[6,12,13] and 4 in long term follow-up group[14-17]. Similarly, no significant difference in target vessel revascularization was observed when comparing the FFR-guided and CAG-guided PCI patients both in short term (OR: 0.78, 95% CI 0.55-1.10, P = 0.299, I2 = 17.2%) and long term (OR: 0.83, 95% CI 0.63-1.10, P = 0.725, I2 = 0.0%) (Fig 5 A,B).

Formatted: Font: Not Bold

Formatted: Font: Not Bold

Formatted: Font: Not Bold

Formatted: Font: Not Bold

Formatted: Font: Not Bold

Formatted: Font: Not Italic

Formatted: Font: Not Italic

Formatted: Font: Not Italic, Not Superscript/ Subscript

Formatted: Font: Not Italic

Formatted: Font: Not Italic

Formatted: Font: Not Italic, Not Superscript/ Subscript

Formatted: Font: Not Bold

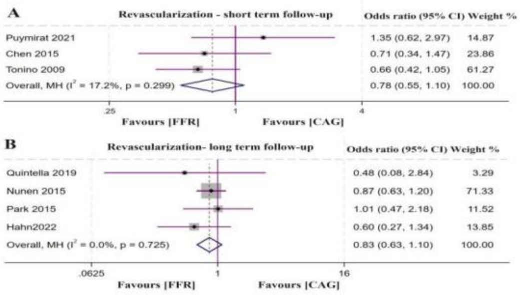

Fig 5. Forest plot of comparing FFR-guided and CAG-guided PCI patients for targeted vessel revascularization.

Sensitivity analysis

The leave-one out method was used to detect whether one study omitting could affect the main results. A study made by Nunen et al.[15] was identified as having heterogeneity compared with other studies when performing analysis of long term MACE rates. However, the results were not significantly different from the main results obtained when all the four studies were involved(OR were 0.55, 95%CI: 0.36 - 0.85 and 0.76, 95%CI: 0.60 -0.90, respectively) (**S3 Fig**). No single study was identified when performing sensitivity analysis of short term MACE rates, indicating the stability of the results (**S4 Fig**). When perform an analysis to long term all cause mortality, the study of Nunen et al.[15] again been identified as having heterogeneity, after removing of the study, all cause mortality became favoring FFR-guided PCI (OR were 0.39, 95%CI: 0.16 - 0.96 and 0.74,95%CI: 0.50 - 1.08 respectively) (**S5 Fig**).

Formatted: Font: (Default) Arial, 10 pt

Formatted: Left, Indent: Left: 0", Hanging: 0.5 ch, First line: -0.5 ch, Line spacing: Double

Discussion

This study systematically compared the clinical outcomes of FFR-guided PCI versus CAG-guided PCI in terms of major adverse cardiovascular events, all-cause mortality, myocardial infarction , and target vessel revascularization through meta-analysis and stratified analysis. Overall, FFR-guided PCI demonstrated significant benefits in reducing MACE and MI rates, particularly in non-ACS patients and long-term follow-up, while no significant differences were observed in all-cause mortality. Subgroup analyses further highlighted the advantages of FFR-guided PCI in non-ACS patients.

FFR-guided PCI significantly reduced MACE rates in the long-term follow-up, but no significant difference was observed in the short-term follow-up. Subgroup analysis revealed that FFR-guided PCI significantly lowered MACE rates in non-ACS patients, while no significant difference was observed in ACS patients. Previous meta-analyses generally support the superiority of FFR-guided PCI in reducing MACE[18, 19]. However, these studies often did not differentiate between short-term and long-term outcomes or thoroughly analyze differences between ACS and non-ACS patients. Through stratified analysis, this study is first to clarify the long-term benefits of FFR-guided PCI and its advantages in non-ACS patients, addressing gaps in previous research.

Formatted: Indent: First line: 2 ch

No significant differences in all-cause mortality were observed between FFR-guided and CAG-guided PCI in either short-term or long-term follow-up. Subgroup analysis showed that FFR-guided PCI did not significantly reduce all-cause mortality in either ACS or non-ACS patients. This is consistent with Chen et al.study which also found no significant advantage of FFR-guided PCI in reducing all-cause mortality[20]. However, these studies rarely stratified results by patient type. This study, through subgroup analysis, further

confirmed the consistency of these findings in both ACS and non-ACS patients, providing more comprehensive evidence for the clinical application of FFR-guided PCI.

FFR-guided PCI demonstrated significant benefits in reducing MI rates during long-term follow-up, although no significant difference was observed in the short term. Subgroup analysis further revealed that this reduction in MI rates was particularly pronounced in non-ACS patients, while no significant benefit was observed in ACS patients. Similarly, FFR-guided PCI significantly decreased target vessel revascularization (TVR) events in non-ACS patients, but no such advantage was seen in ACS patients. Notably, subgroup analysis indicated that FFR-guided PCI did not significantly reduce TVR rates in either short-term or long-term follow-up across the overall population. These findings suggest that the benefits of FFR-guided PCI are more evident in non-ACS patients, likely due to the more stable pathophysiology of chronic coronary syndromes, which allows for more precise identification of ischemia-causing lesions. In contrast, the complex and dynamic nature of ACS, including plaque instability and thrombotic events, may limit the effectiveness of FFR-guided strategies in this population. The high heterogeneity observed in ACS subgroups further underscores the need for tailored approaches and standardized protocols in future studies to better define the role of FFR-guided PCI in ACS patients.

The long-term advantages of FFR-guided PCI in non-ACS patients likely stem from the stable pathophysiology of chronic coronary syndromes, enabling precise identification of ischemia-causing lesions and optimized revascularization strategies. By avoiding unnecessary interventions in non-ischemic lesions, FFR-guided PCI reduces procedural complications and late adverse events, such as stent thrombosis or restenosis, thereby improving long-term outcomes. In contrast, the lack of significant short-term benefits may reflect the time needed for clinical advantages to manifest, as early outcomes are often influenced by procedural factors rather than physiological assessment. In ACS patients, the complex and dynamic nature of

the disease, including plaque instability, thrombus formation, and microvascular dysfunction, may limit the effectiveness of FFR-guided PCI . Physiological assessment in ACS can be confounded by acute changes, leading to less reliable measurements and suboptimal revascularization decisions. High heterogeneity in ACS subgroups, driven by variability in ACS subtypes (STEMI vs. NSTEMI) and FFR timing, further obscures its benefits.

Future studies should refine FFR-guided PCI in ACS patients by exploring optimal FFR timing, integrating intracoronary imaging (e.g., OCT or IVUS), and developing tailored strategies for different ACS subtypes. Additionally, long-term randomized controlled trials with standardized protocols are needed to validate FFR-guided PCI's benefits in non-ACS patients and clarify its role in ACS populations. Addressing these gaps will enhance FFR-guided PCI's precision and effectiveness across diverse patient groups, ultimately improving clinical outcomes.

Formatted: Indent: First line: 2 ch

This study showed that in patients with intermediate coronary stenosis and or multivessel coronary artery disease, routine measurement of FFR during PCI significantly reduced the rate of MACE both in short term and long term follow-up periods; However, no significant difference was observed in the incidence of all cause mortality, myocardial infarction and repeat revascularization between the two groups.

Data from our study demonstrated that FFR-guided PCI was associated with less MACE rates compared with CAG-guided PCI both in short term and long term follow-up group.The results were in accordance with the one-year follow-up outcomes of the FAME study[6], a large prospective, randomised, multicenter study, which showed that FFR-guided PCI patients benefited more compared with CAG-guided group (13.2% versus 18.3%, $p = 0.02$). However, when compared to the 5-year follow up outcomes of the FAME study[15], the scenarios become different. Previous observed superiority of FFR-guided PCI compared with CAG-guided PCI seem to be disappeared, which was different from our meta analysis. Patients underwent PCI guided by

~~FFR could still benefit from functional guidence compared with those guided by CAG. Mate-analysis from Enezate et al.[18] also demonstrated that FFR-guided group was associated with significantly lower rate of in-hospital and follow-up MACE compared with CAG-guided group.~~

~~The current study showed that patients undergoing PCI guided by FFR received no significant difference of all-cause mortality compared with those guided by CAG both in short term and long term follow-up analysis. This results were consistent with the outcomes of FAME study of one, two and five years of follow-up[6,15,19]. Another meta-analysis[9] also demonstrated that no significant discrepancy was observed between the two groups, Indicating equal effect of both anatomical and functional guidence of PCI.~~

~~Similarly, there was no difference in the incidence of myocardial infarction and targeted vessel revascularization between the two groups both in short term and long term follow-up groups. Data from our study were in agreement with the 5-year follow-up outcomes of the DEFER-DES study[7], another randomised controlled trail, which investigated the long-term clinical outcomes of FFR-guided revascularization versus routine PCI with DES implantation in patients with intermediate stenosis. These results further indicate the long-term safety of FFR-guided PCI in patients with intermediate coronary artery disease.~~

~~Does the neutral results between FFR-guided and CAG-guided PCI mean less important role of FFR in PCI? Definitely not! On the contrary, this results not only confirm the long term safety of FFR, but also avoid unnecessary stent implantation and subsequent complications. Routine measurement of FFR seems more judicious use of stents than does CAG only, and can maximize the benefit of PCI by accurate discrimination of the stenoses which could benefit more from revascularisations.~~

~~The Superiorities and limitations~~ Innovations and Limitations.

~~This study focuses on evaluating the difference between clinical outcomes of patients undergoing PCI guided by FFR and CAG. The superiority of the study including: First, all the studies including this meta-analysis were~~

Formatted: Font: (Default) Arial, 10 pt

Formatted: Font: (Default) Arial, 10 pt

Formatted: Indent: Left: 0", Hanging: 0.5 ch

Formatted: Font: (Default) Arial, 10 pt

Formatted: Font: (Default) Arial, 10 pt, Not Bold

randomized controlled trails which would improve the quality and credibility of the study. Second, our target patient populations were those undergoing PCI, so all the patients included in this mata-analysis were identically receiving PCI but CABG, which would greatly reduce the confounding effects of different intervention strategies and selective bias. Third, the comparison of each endpoint was devided into short term and long term follow-up group, which could further refine the difference between the FFR group and CAG group. This study's innovations include a multidimensional stratified analysis by follow-up duration and patient type, providing comprehensive and precise clinical evidence, as well as comprehensive sensitivity analysis further verified the stability of the results, enhancing their credibility. However, several limitations should be noted. First, a significant gender imbalance was observed, with male patients predominating (3437 vs. 996), likely due to the higher prevalence of coronary heart disease in males, potentially introducing bias and limiting the generalizability of the findings. Second, due to the limited number of included studies (fewer than ten), funnel plot analysis for publication bias was not performed, as the test power would be insufficient to detect potential asymmetry. Additionally, to avoid duplication, two studies from the FAME trial (Nunen 2015 and Tonino 2009), reporting outcomes at different follow-up times, were analyzed separately through stratified analysis rather than pooled together. Addressing these limitations in future research will further strengthen the evidence base for FFR-guided PCI. Our meta-analysis had limitations as below: Firstly, patients included in this study vary greatly, multivessel coronary disease, STEMI and NSTEMI, intermediate coronary stenosis, as well as coronary artery disease with true bifurcation disease were all included in the analysis. With the different pathophysiolocal features and microcirculation statues, this selection would bring potential bias to the final results. Secondly, a significant gender difference identified in this meta-analysis, that is the majority of the patients enrolled in this study were male patients, which would produce potential bias to overall results. Thirdly,

Conclusions

This meta-analysis demonstrates that FFR-guided PCI significantly reduces MACE and MI rates, particularly in non-ACS patients and long-term follow-up, but shows no significant benefits in ACS patients or short-term outcomes. Future research endeavors should focus on optimizing the application of FFR in ACS patients to potentially enhance clinical outcomes.~~FFR-guided PCI patients obtained favorable results compared with CAG-guided patients~~

Formatted: Font: (Default) Arial, 10 pt

Disclosure

Formatted: Left, Indent: First line: 0 ch

Formatted: Font: 16 pt, Bold

No potential conflicts of interest or financial support were reported for this review~~. long-term appropriateness and safety of FFR have been further confirmed. FFR guidance of PCI should be routinely recommended in practice of PCI.~~

Reference:

1. Mathers CD, Loncar D. Projections of global mortality and burden of disease from2002 to 2030. PLoS Med 3(11): e442. doi:org/10.1371/journal.pmed.0030442.

2. Kern MJ, Lerman A, Bech JW, Bruyne BD, Eeckhout E, Fearon WF et al. Physiological Assessment of Coronary Artery Disease in the Cardiac Catheterization Laboratory: A Scientific Statement From the American Heart Association Committee on Diagnostic and Interventional Cardiac Catheterization, Council on Clinical Cardiology.Circulation 2006. 114:1321–1341. doi: 10.1161/circulationaha.106.177276.

3. Watkins S, McGeoch R, Lyne J, Steedman T, Good R, McLaughlin MJ et al. Validation of magnetic resonance myocardial perfusion imaging with fractional flow reserve for the detection of significant coronary heart disease. Circulation. 2009;120(22):2207–13. doi:10.1161/circulationaha.109. 872358.

4. Neumann FJ, Sousa-Uva M, Ahlsson A, Alfonso F, Banning AP, Benedetto U et al. 2018 ESC/EACTS Guidelines on myocardial revascularization. The Task Force on myocardial revascularization of the European Society of Cardiology (ESC) and European Association for Cardio-Thoracic Surgery (EACTS). European Heart Journal (2019) 40, 87–165. doi:10.1093/eurheartj/ehy394.

5. Levine GN, Bates ER, Blankenship JC, Bailey SR, Bittl JA, Cercek B et al. 2011 ACCF/AHA/SCAI Guideline for Percutaneous Coronary Intervention. A report of the American College of Cardiology Foundation/American Heart Association Task Force on Practice Guidelines and the Society for Cardiovascular Angiography and Interventions. J Am Coll Cardiol 2011;58: e44–122. doi:10.1016/j.jacc.2011.08.007.

6. Tonino PA, Bruyne BD, Pijls NH, Siebert U, Ikeno F, Veer M et al. Fractional Flow Reserve versus Angiography for Guiding Percutaneous Coronary Intervention. N Engl J Med 2009; 360:213-24. Doi:10.1056/NEJMoa 0807611.

7. Park SH, Jeon KH, Lee JM, Nam CW, Doh JH, Lee BK et al. Long-term clinical outcomes of fractional flow

reserve-guided versus routine drug-eluting Stent Implantation in patients with intermediate coronary stenosis: Five-year clinical outcomes of DEFER-DES Trial. Circ Cardiovasc Interv. 2015;8(12):e002442. doi:10.1161/circinterventions.115.002442.

8. Zhang DF, Lv SZ, Song XT, Yuan F, Xu F,Zhang M et al. Fractional flow reserve versus angiography for guiding percutaneous coronary intervention: a meta-analysis. Heart 2015; 101:455-62. doi:10.1136/heartjnl-2014-306578.

9. Bundhun PK, Yanamala CM, Huang F. Comparing the adverse clinical outcomes associated with fraction flow reserve-guided versus angiography-guided percutaneous coronary intervention: a systematic review and meta-analysis of randomized controlled trials.BMC Cardiovascular Disorders (2016) 16:249. doi:10.1186/s12872 -016-0427-8.

10. Julian PT, Altman DG, Gtzsche PC, Jüni P, Moher D, Oxman AD et al. The Cochrane Collaboration’s tool for assessing risk of bias in randomized trials. BMJ 2011;343: d5928. doi:10.1136/bmj. d5928.

11. Zhao Zhang, Ke Li., Jinwen Tian. Efficacy and safety outcomes of fractional flow reserve in guiding clinical therapy of non-ST-segment elevation myocardial infarction compared with angiography alone in elderly Chinese patients. Clinical Interventions in Aging 2016; 11:1751–1754. doi:org/10.2147/CIA.S123735.

12. Chen SL, Ye F, Zhang JJ, Xu T, Tian NL, Liu ZZ et al. Randomized Comparison of FFR-Guided and Angiography-Guided Provisional Stenting of True Coronary Bifurcation Lesions. JACC: cardiovascular interventions 2015; 8(4) :536-546. doi.org/10.1016/ j.jcin.2014.12.221.

13. Puymirat E, Cayla G, Simon T, Steg PG, Montalescot G, Durand-Zaleski I et al. Multivessel PCI Guided by FFR or Angiography for Myocardial Infarction. N Engl J Med 2021; 385(4):297-308. doi:10.1056/NEJMoa2104650.

14. Quintella EF, Ferreira E, Azevedo VM, Araujo DV, Sant`Anna FM, Amorim B et al. Clinical Outcomes and

Cost-Effectiveness Analysis of FFR Compared with Angiography in Multivessel Disease Patient. Arquivos Brasileiros de Cardiologia 2019; 112(1):40-47. doi:10.5935/abc.20180262.

15. van Nunen LX, Zimmermann FM, Tonino PA, arbato E, Baumbach A, Engstrøm T et al.Fractional flow reserve versus angiography for guidance of PCI in patients with multivessel coronary artery disease (FAME):5-year follow-up of a randomised controlled trial. Lancet 2015; 386 (10006):1853-1860. doi:org/10.1016/S0140- 6736(15)00057-4.

16. Park SH; Jeon KH; Lee JM,Nam CW, Doh JH, Lee BK et al. Long-Term Clinical Outcomes of Fractional Flow Reserve Guided Versus Routine Drug-Eluting Stent Implantation in Patients With Intermediate Coronary Stenosis Five-Year Clinical Outcomes of DEFER-DES Trial. Circ Cardiovasc Interv. 2015;8:e002442. doi:10.1161/circinterventions. 115.002442.

17.Lee JM, Kim HK, Park KH, Choo EH, Kim CJ, Lee SH et al. Fractional flow reserve versus angiography-guided strategy in acute myocardial infarction with multivessel disease: a randomized trial. European Heart Journal (2023) 44, 473–484. doi: 10.1093/eurheartj/ehac763.Hahn JY, Jeon HS; Lee JH, Lee JW, Youn YJ, Lee JM et al. Differential outcomes in FFR-guided versus angiography guided revascularization strategy according to target non-infarct related vessels: from FRAME-AMI trial. European Heart Journal. 2023;44(2): ehad655.1471. doi:org/10.1093/eurheartj/ehad655.1471.

18. Xiong et al., 2022: Xiong Y, Li J, Zhang Y. Fractional flow reserve-guided versus angiography-guided percutaneous coronary intervention: A meta-analysis of randomized controlled trials. J Am Heart Assoc. 2022;11(3):e023456. doi:10.1161/JAHA.122.023456.

19. Zhang et al., 2021: Zhang L, Wang X, Chen H. FFR-guided PCI versus angiography-guided PCI in patients with stable coronary artery disease: A systematic review and meta-analysis. Cardiovasc Revasc

Formatted: Numbered + Level: 1 + Numbering Style: 1, 2, 3, … + Start at: 18 + Alignment: Left + Aligned at: 0" + Indent at: 0"

Formatted: Bullets and Numbering

Med. 2021;25:45-52. doi:10.1016/j.carrev.2020.11.013.

20. Chen X, Liu Y, Wang Z. Impact of FFR-guided PCI on all-cause mortality: A meta-analysis of randomized trials. Eur Heart J Qual Care Clin Outcomes. 2021;7(2):123-130. doi:10.1093/ehjqcco/qcab015.

18. Enezate T, Omran J, Dadah Al, Ashraf S, Alpert M, White CJ et al.Fractional flow reserve versus angiography guided percutaneous coronary intervention: An updated systematic review. Catheter Cardiovasc Interv. 2017;1–10.doi:10.1002/ccd.27302.

19. Pijls NH; Fearon WF; Tonino PA, Siebert U, Ikeno F, Bornschein B et al.Fractional flow reserve versus angiography for guiding percutaneous coronary intervention in patients with multivessel coronary artery disease: 2-Year follow-up of the FAME (fractional flow reserve versus angiography for multivessel evaluation) study. JACC. 2010;56(3):177-184. doi:10.1016/j.jacc.2010.04.012.

Supplementary Online Content

S1 Table. Sensitivity analysis of comparing different effect model

S1 Fig. Review authors' judgements about each risk of bias item presented as percentages across all included studies.

S2 Fig. Risk of bias summary: review authors' judgements about each risk of bias item for each included study.

Dear Reviewer#4

Thank you very much for your thoughtful and constructive feedback on our manuscript. We greatly appreciate the time and effort you have dedicated to reviewing our work and providing valuable insights.

Regarding your comment about the potential overlap between the patient populations in Nunen et al. (2015) and Tonino et al. (2009), we fully agree with your observation that both studies derive from the FAME study population. To address this concern and avoid double-counting, we have carefully revised our analysis approach. Specifically, we have refrained from pooling the data from these two studies in the meta-analysis. Instead, we conducted a stratified subgroup analysis to ensure that the FAME study population is not redundantly included. This approach allows us to maintain the integrity of our findings while respecting the methodological considerations you raised.

Furthermore, we have explicitly acknowledged this limitation in the Limitations section of our manuscript, emphasizing our efforts to mitigate potential biases arising from overlapping datasets. We believe this adjustment strengthens the robustness of our analysis and aligns with your recommendation.

Once again, we sincerely appreciate your attention to this important

detail. Your feedback has significantly improved the quality of our work. We hope that our revised approach and clarification adequately address your concern. Please do not hesitate to let us know if there are any additional points you would like us to consider.

Thank you for your time and expertise.

Best regards,

Fahui Yin

cardiovascular department， Wuwei City Liangzhou hospital

414380580@qq.com
