# Supplementary material for: Updated meta-analysis of fractional flow reserve versus coronary angiography for guiding percutaneous coronary intervention
Source: PLoS One. 2025 Oct 9;20(10):e0334019. doi: 10.1371/journal.pone.0334019 (PMC12510650; doi:10.1371/journal.pone.0334019)
Supplement: Attachment — Submitted filename: Response to Reviewers.docx [file pone.0334019.s008.docx]

**A Rebuttal letter**

Dear editors and reviewers:

Tank you for your letter and the reviewers’ comments concerning our manuscript entitled “Updated Meta-Analysis of Fractional Flow Reserve versus Coronary Angiography for Guiding Percutaneous Coronary Intervention”. These comments are very important and highly valuable for me to revise the manuscript and improve the quality of the meta analysis. We have read these comments carefully and modified related issues. We hope the revised version of manuscript could meet the standard of the journal and the approve of the reviewers. The revised portion were marked in red in the manuscript. The point to point responds to the comments of the editors and three reviewers were as following:

Academic editor:

**Comment 1:** 1. Please ensure that your manuscript meets PLOS ONE's style requirements, including those for file naming. The PLOS ONE style templates can be found at:

**Response 1:** According to the requirements of PLOS ONE, we throughly checked up the manuscript and modified those which did not fulfill the requirements of the journal.

2. **Comment 2:** Please include captions for your Supporting Information files at the end of your manuscript, and update any in-text citations to match accordingly. Please see our Supporting Information guidelines for more information:

**Response 2:** According to your advice, we included captions for Supporting Information files at the end of the manuscript.

**Comments 3(a):** As required by our policy on Data Availability, please ensure your manuscript or supplementary information includes the following:

A numbered table of all studies identified in the literature search, including those that were excluded from the analyses.

**Response3(a):** According to your advice, we uploaded the numbered table of included and excluded studies identified in the literature search.

**Comments 3(b):** For every excluded study, the table should list the reason(s) for exclusion.

**Response3(b):** According to your advice, the reasons for exclusion were included in the table.

**Comments 3(c):**If any of the included studies are unpublished, include a link (URL) to the primary source or detailed information about how the content can be accessed.

**Response3(c):** All the included studies have been published, and the full text have been obtained.

**Comments 3(d):**A table of all data extracted from the primary research sources for the systematic review and/or meta-analysis. The table must include the following information for each study:

Name of data extractors and date of data extraction

**Response3(d):** A table of all data extracted from the primary research sources for the systematic review were uploaded as supplement materials, and the name of data extractors and date of data extraction were also included.

**Reviewer#1**

**Comment 1:** Abbreviations should be explained in the main body of the text when they first appear.

**Response 1:** We are very sorry for our negligence of the abbreviations. According to your comments, we made correction of the manuscript and checked the full body of the paper.

**Comment 2:** The DEFER study is the first FFR vs angio-guided PCI study (not FAME) and should be included in the analysis.

**Response 2:** Indeed, the DEFER study is the first randomised controlled study comparing FFR-guided and angiography-guided PCI. which was published in 2001, all the participants enrolled the study were undergone coronary balloon dilatation. However, the outcomes of the the DEFER study were event-free survival and the percentage of patients free from angina which were not our predefined outcomes. The DEFER-DES Trial was a prospective, randomized study conducted in 6 university hospitals in Korea, and the later one satisfy our inclusion criteria.

**Comment 3:** This study compared FFR vs angio-guided PCI in NSTEMI and should be included in the analysis.

**Response 3:** We carefully read the article entitled“Total versus staged versus functional revascularization in NSTEACS patients with multivessel disease” recommended by the reviewer, This RCT was a three-armed trail comparing total revascularization group (total group), staged revascularization group (staged group), and functional revascularization group of NSTEACS patients. Which were different from our enrolled two-armed RCT studies. Perhaps the pooled analysis of two and three armed RCT do exist in previous meta-analysis, however, this kind of selective meta analysis seemed inappropriate, and probably bring certain bias to the overall results of the meta analysis.

**Comment 4:** FLOWER MI has 1 year MACE data and should be included in the analysis.

**Response 4:** According to your suggestion, we carefully read the article and extracted the data related MACE. Finally we remade the meta analysis.

**Comment 5:** Why is the main study for FRAME AMI not quoted? Instead a subgroup analysis from a conference abstract was quoted in its place. Please use the FRAME AMI main study data and reference: [https://pubmed.ncbi.nlm.nih.gov/36540034/](https://pubmed.ncbi.nlm.nih.gov/36540034/)

**Response 5:** I sincerely express the great appreciation to the reviewer #1 for providing me the publication version of FRAME AMI study which I searched for a lot of time. The main study of FRAME AMI would great improve the quality of the present meta-analysis. We have replaced the conference abstract with the main study in reference, the other places such as risk of bias assessment were also made correction in the manuscript.

**Comment 6:** In your first results paragraph comparing MACE rates of FFR-guided PCI vs angio-guided PCI, you stated that FFR PCI was associated with significant reduction of MACE during short term and long terms, with both 95% confidence intervals appropriately below 1.00, but the p values are non-significant (0.476 and 0.126)- is this an error?

**Response 6:** When we perform the meta analysis with the software of stata 18.0 version, the outcomes of P value usually is responsible for the heterogeneity test. When I^2^ is great than 50%,the value of P will be less than 0.05,which can been found in the forest plot ; when I^2^ less than 50%, the P value will become greater than 0.05. In the revised manuscript, we change the P value of the each outcomes, which is responsible for the total combined effect size.However, this value cannot been found in forest plot.

**Comment 7:** You should consider doing a subgroup analysis of ACS vs non ACS RCTs.

**Respomse 7:** According to your advice, we performed a subgroup analysis based on patients categories (ACS vs non-ACS) .

**Comment 8:** Is the meta analysis done with fixed or random effects? Please state.

**Response 8:** Owing to the less value of I^2^, no more than 50%, the effects model was used fixed effects model.In the sensitivity analysis, we perform both fixed and random effects model to compare the total effects of the two models.

**Comment 9:** Please consider doing a meta-regression analysis; Is there evidence of publication bias?

**Comment 9:** In this meta analysis, we performed subgroup analysis to detect the origin of the heterogeneity among enrolled study, which is based on follow-up durations and patients categories. Taking consideration of the similarity of the two methods, so the meta-regression analysis was not made in this study. Funnel plot was usually used to detect if there is publication bias existing, however, when the enrolled studies were less than 10, the power of funnel plot was unable to detect the real asymmetry of the enrollled studies. only 8 studies included in our study, so we did not perform the funnel plot, the publication bias probably been existing. We described the above in the limitation section.

**Comment 10:** There are many spelling mistakes- please check the manuscript thoroughly for spelling errors: Eg: Your title should be "meta-analysis" not "mata-analysis"

**Response 10:** We are very sorry for the spelling errors, Thank your kind suggestion, we have made the corrections of the spelling mistakes,and carefully read and checked the manuscript before submission.

Reviewer#2

**Comment 1:** Gender Bias:

With the majority of patients being male, the authors should explore the potential impact of this imbalance on generalizability and explicitly acknowledge it in the limitations.

**Response 1:** In the limitation section, we point out the gender bias in the meta analysis, and the potential reason might been associated with an higher morbidity rate of coronary heart disease in male patients, and this gender discrepancy could problely produce potential bias to the overall results.

**Comment 2:** Comparison with Existing Meta-Analyses:

Including a more detailed discussion comparing the current findings with similar meta-analyses would provide important context and help highlight the novelty of the study’s conclusions.

**Response 2:** In the discussion section, we compared our study with previous published meta-analysis,we did a subgroup analysis which demonstrated that FFR-guided PCI was superior to CAG-guided PCI in long term follow up subgroup and non-ACS subgroup, and no significant difference was observed in ACS group. We also explore the potential reasons from pathophysiologic aspects. This conclusion could not been found in previous published meta analysis and should be the novelty of the present meta analysis.

**Comment 3:** In-depth discussion on pathophysiological factors:
Many pathophysiological factors can influence the treatment efficacy of PCA. Especially, microvascular dysfunction can significantly influence the treatment efficacy and prognosis but is challenging to evaluate (10.1631/jzus.B2100425). An in-depth discussion on other pathophysiological factors is expected.

**Response 3:** Indeed, many pathophysiological factors can influence the efficacy of PCI. In this meta analysis we found that FFR-guided PCI was not superior to CAG-guided PCI in ACS subgroup analysis. And try to explore the potential pathophysiologic characteristics in ACS subgroup patients in the discussion section.

**Comment 4(a):** Improve Statistical Rigor and Reporting:
a. Clarify Model Selection: The manuscript should specify when and why a particular model (e.g., fixed-effect or random-effect) was chosen. A fixed-effect model assumes that all studies estimate the same effect size, which works well if heterogeneity is low (I² < 50%). In contrast, a random-effect model accounts for between-study variability and is more appropriate when heterogeneity is substantial (I² > 50%). For example, the use of a random-effect model for long-term MI outcomes (I² = 71.8%) should be justified to improve transparency.

**Response 4(a):** Yes, we described the model selection in the statistical analysis section.
**Comment 4(b):** Interpret Sensitivity Analyses: Although the manuscript mentions a leave-one-out sensitivity analysis, further explanation is needed on why certain studies (e.g., Nunen et al.) introduced heterogeneity and how their exclusion influenced the overall results. This would provide deeper insights into the robustness of the findings and clarify the sources of variation.

**Response 4(b):** In this revised manuscript we changed the method of sensitivity analysis, by comparing the total effect value of the fixed and random model. As we all known that the fixed effect model usually gives more weight to the large sample study, while random effect model gives more weight to small sample study. By comparing the two model could find if small sample study has an impact on the total effects value.
**Comment 4(c):** Assess Publication Bias: The manuscript does not address potential publication bias, which can distort meta-analytic results. Including funnel plots and performing statistical tests (e.g., Egger’s regression test) would improve rigor and ensure that the findings are not skewed by selective reporting.

**Response 4(c):** In the limitation section we described the reason why we did not perform the funnel plot. Because only 8studies enrolled into the meta analysis , and the funnel plot is unable to detect the real asymmetry of the enrollled studies, So the publication bias of the present meta-analysis probably been existing.

Reviewer #3:

**Comment 1:** Line 1: The title of the review is misspelled. Please change from “mata-analysis”

**Response 1:** The mistake have been revised,and we carefully check the full manuscript before re-submission to avoid spelling mistakes.

**Comment 2:** Line 8: What does “were medical searched” refer to?

**Response 2:** “Medical search” is an official explanation of searching medical data bases by a set of strict methods, such as predefined search terms, searching methods et al.

**Comment 3:** Line 14-17: Check spacing and typography when specifying results of meta-analysis. 95% CI, P-values etc. should be written appropriately to avoid confusion.

**Response 3:** We carefully check the spacing and typography before re-submission.

**Comment 4:** Line 21: short and long term follow-up seems to be repeated.

**Response 4:** We have done modifications.

**Comment 5:** Line 32-44: It is important to clearly explain the research gap and why you expect this gap to exist. From what has been written it is not clear why FFR may be superior to angiography, and if guidelines are clear on the gold standard approach what this review will add.

**Response 5:** In the introduction section, we described that coronary angiography estimated the coronary stenosis by vision assessment, which usually over-estimates or under-estimates the severity of the intermediate coronary stenosis. FFR could provide an scientific assessment of the target vessel from functional aspect. So it is recommended by many guidelines.the purpose of this meta analysis is to further compare FFR and CAG in guiding PCI.

**Comment 6:** Line 50-55: Just because other reviews did not compare RCTs does not mean their results are not significant, as evidenced by guidelines agreeing with them and recommending FFR. When doing a redo meta-analysis it is important to justify what will be different to the previous ones, and why there is still a research need.

**Response 6:** Indeed, the words like “the quality cannot reach high level” seemed arbitrary and inappropriate.we delete the phrase.

Methods:

**Comment 7:** Line 60: Was this protocol conducted in line with PRISMA statement? Was there a pre-specified protocol published on a repository such as PROSPERO? It is important to specify whether such methodological standards were met.

**Response 7:** Yes, the protocol was conducted in line with PRISMA statement. A detailed PRISMA statement was attached in the supplement materials. We had ever tried to registered the protocol in official website of PROSPERO, and submitted the materials on the website, however, the website seemed not work well and received on reply. It is really a pity for me and the present study.

**Comment 8:** Line 63: Mention whether the search strategy is available to the reader

**Response 8:** Yes, we have the screenshot of the whole process of medical searching. I will upload the materials in the re-submission.

**Comment 9:** Line 67: This is vague, were any specific types of patients excluded based on comorbidities or indication for PCI?

**Response 9:** Because the objects of meta analysis are those published studies. So it is difficult to identify if the patients excluded based on comorbidities, however, those patients who had been received CABG excluded from this meta analysis.

**Comment 10:** Line 68: Does this mean you will aggregate results for all types of patients as long as they all receive PCI? Will this reduce generalisability to your results and did you plan to do a subgroup analysis?

**Response 10:** No, our inclusion criteria were: (1) Randomised controlled trails (RCTs) ; (2) Comparing FFR-guided and angiography-guided PCI; (3) Reporting at least one of the following outcomes, including MACEs, all-cause mortality, myocardial infarction, and target vessel revascularization. This three requirements must been fully met. Maybe our study inclusion criteria seemed strictly and would exclude many previous published famous studies from our meta analysis, however, with accumulating the evidence of FFR practice, We now could better compare FFR and CAG in specific type of patients, such as receiving PCI patients, meanwhile, we did subgroup analysis based on follow up duration and patients categories which could better compare the two methods under different situations.

**Comment 11:** Line 74-75: Why is short-term 1 year and beyond this long-term? In the context of PCI and intervention, typically 5 and 10 year results are thought to be long-term.

**Response 11:** Firstly, In daily practice, the one year follow up is very important for patients undergoing PCI; Secondly, Many studies published had been used one year as the follow up period. According to the follow up periods of the studies we have been searched, we divided them into short term and long term period.

**Comment 12:** Line 83: Please clarify which clinical outcomes and baseline characteristics were extracted

**Response 12:** The detailed clinical outcomes and baseline characteristics can been found in table 1.

**Comment 13:** Line 84: Why RoB and not RoB2?

**Response 13:**Indeed, ROB2 is more valuable in bias control compared with previous RoB, According to your advice, we uesd ROB2 instead of ROB in the revised manuscript.

**Comment 14:** Line 88: PRISMA refers not only to statistical analysis, move this to the start of methods.

**Response 14:**According to your advice, we have moved the PRISMA to the methods section.

**Comment 15:** Line 89-95: This section describes what you did in the meta-analysis but fails to adequately describe anything else regarding your work. How did you process and present the data for your baseline characteristics? How did you determine which variables would be meta-analysed? How did you determine whether heterogeneity was significant? How did you assess for publication bias or quality of evidence? These questions and the overall purpose of the analysis are not clear from what you have written.

**Response 15:** Baseline characteristics were present in table 1, we added the type of variables and the methods for meta-analysed. Meanwhile, subgroup analysis, heterogeneity test, sensitivity analysis were also clarified in this section.

**Comment 16:** Line 122: What does the range (14.2-36.4%) with diabetes indicate and how was this calculated? Please clarify this in the methods and update results section of manuscript to match.

**Response 16:** The range of (14.2-36.4%) with diabetes indicate the lowest rate and the highest rate of diabetes. It is calculated by using the number of patients with diabetes divided by the number to total patients in each group. However, these data seems inappropriate to describe the difference between the two groups. So we delete these data in the revised manuscript.

**Comment 17:** Line 124: This is the first mention of the protocol, what is this and where can it be found? Please elaborate in results.

**Response 17:** Probably this is a wrong description. According the design of the present, we did a subgroup analysis based on follow up duration and patients categories.

**Comment 18:**Line 125: The length of follow-up is not consistent, will results be aggregated across multiple studies with varying length of follow-up? Please report what this average follow-up length is for the reader also.

**Response 18:** Yes, the length of follow-up is not consistent among the different studies. It is difficult to calculate the average follow up length among different studies, but can provide a certain range of follow up duration according to the included studies. We have added it in the results section.

**Comment 19:** Line 148: Please mention how many patients were included in each analysis. This would benefit reader for all meta-analysis conducted.

**Response 19:** According to your advice,we have added it in the revised manuscript.

**Comment 20:** Line 149: PCI has a lower rate of MACE but the p-value is 0.476? This is not significant please verify the number is correct across all meta-analysis.

**Response 20:**This question also raised by the other two reviewers. When we perform the meta analysis with the software of stata 18.0 version, the outcomes of P value usually is responsible for the heterogeneity test. When I2 is great than 50%, the value of P will be less than 0.05, which can been found in the forest plot ; when I2 less than 50%, the P value will become greater than 0.05. In the revised manuscript, we change the P value of the each outcomes, which is responsible for the total combined effect size.

**Comment 21:** Line 178: What was the significance of this heterogeneity and how was it determined?

**Response 21:** In the revised manuscript, we remade the meta analysis of MACE by adding an study with MACE. However, when we performed subgroup analysis of follow up duration, the heterogeneity still been considered existing because of great value of I2. so random effect model was made.

**Comment 22-34:** Discussion section line199-line 236.

Line 199: Why would you refer to a trial included within your meta-analysis to justify your results are in line with previously established results?

Line 207-208: It would be pertinent to explain how MACE is reduced in FFR when mortality, TVR, and MI were the same in both groups, both clinically and within your study results.

Line 209: But you only included the FAME study at 1-year of follow-up? When there was longer data available? What was the reason for this and mention in methods how you approached trials reporting multiple sets of results (i.e. from different time periods or subgroups).

Line 210: What was the result of this meta-analysis and how was your study similar/different? Compare and contrast number of patients and meta-analysis results more thoroughly.

Line 214: DEFER-DES is included within your meta-analysis, of course your results will match what they did. Please explain why your result was why it was the case and how conducting your meta-analysis added to the result.

Line 219: It is not appropriate to use rhetoric such as “Definitely not!” in a manuscript.

Line 220: Is this not what guidelines have been saying already based on previous analysis? How does your meta-analysis contribute towards existing understanding of FFR and its usage in clinical practice?

Line 222: This section of the manuscript would benefit from significant addition, as it is unclear as to why FFR is superior to CAG clinically from what has been written thus far.

Line 228: Having all the patients receive PCI does not reduce confounding; it simply makes them appropriate to compare since they are all undergoing the same intervention.

Line 229-230: But the 1-year cutoff was used arbitrarily and is not in line with previous literature (see my comment from earlier)

Line 231: This is a limitation of the inclusion criteria which can be rectified – limitations of the study ideally are things out of your control.

Line 234: Has this been justified before? If so, please reference.

Line 236: This manuscript says “thirdly” abruptly. Please complete this section.

**Response 22-34:** According to your advice, we have deleted some of those inappropriate descriptions. Additionally, owing to adding subgroup analysis according to ACS and non-ACS, we found that FFR-guided PCI patients obtained favorable results compared with CAG-guided patients on MACE, all cause mortality, MI and revascularizations, especially in long term follow-up subgroup and non-ASC subgroup.no significant difference was observed in ACS patients. Compared with previous version of manuscript, the revised one refined the comparison between FFR and CAG in different patient category.So we modified and rewrote the discussion section of the revised manuscript.

**Comment 35:** Line 240: Expand on the context of the result and how this study aids practice.

**Response 35:** This study found that FFR-guided PCI patients obtained favorable results compared with CAG-guided patients on MACE, all cause mortality, MI and revascularizations, especially in long term follow-up subgroup and non-ASC subgroup.no significant difference was observed in ACS patients. so FFR should be avoided in ACS patients, which can be found in conclusion section.

In conclusion, we have thoroughly addressed all the concerns raised by the reviewers. We are deeply grateful to the reviewers for their careful consideration and insightful comments. Their expertise and dedication have greatly enhanced the quality of our work. We also appreciate the efforts of the editorial team in facilitating this review process.

We look forward to the reviewers' further assessment of our revised manuscript. We are confident that the changes we have made will meet the publication standards of your esteemed journal.

Thank you once again for your time and efforts.

Best regards,

Fahui Yin

Liangzhou Hospital, Wuwei City

[414380580@qq.com](mailto:414380580@qq.com)

Dec 9 2024
