# Supplementary material for: Updated meta-analysis of fractional flow reserve versus coronary angiography for guiding percutaneous coronary intervention
Source: PLoS One. 2025 Oct 9;20(10):e0334019. doi: 10.1371/journal.pone.0334019 (PMC12510650; doi:10.1371/journal.pone.0334019)
Supplement: Attachment — Submitted filename: Response to reviewer #4.docx [file pone.0334019.s009.docx]

Dear Reviewer#4

Thank you very much for your thoughtful and constructive feedback on our manuscript. We greatly appreciate the time and effort you have dedicated to reviewing our work and providing valuable insights.

Regarding your comment about the potential overlap between the patient populations in Nunen et al. (2015) and Tonino et al. (2009), we fully agree with your observation that both studies derive from the FAME study population. To address this concern and avoid double-counting, we have carefully revised our analysis approach. Specifically, we have refrained from pooling the data from these two studies in the meta-analysis. Instead, we conducted a stratified subgroup analysis to ensure that the FAME study population is not redundantly included. This approach allows us to maintain the integrity of our findings while respecting the methodological considerations you raised.

Furthermore, we have explicitly acknowledged this limitation in the Limitations section of our manuscript, emphasizing our efforts to mitigate potential biases arising from overlapping datasets. We believe this adjustment strengthens the robustness of our analysis and aligns with your recommendation.

Once again, we sincerely appreciate your attention to this important detail. Your feedback has significantly improved the quality of our work. We hope that our revised approach and clarification adequately address your concern. Please do not hesitate to let us know if there are any additional points you would like us to consider.

Thank you for your time and expertise.

Best regards,
Fahui Yin
cardiovascular department， Wuwei City Liangzhou hospital
414380580@qq.com
