# Supplementary material for: Updated meta-analysis of fractional flow reserve versus coronary angiography for guiding percutaneous coronary intervention
Source: PLoS One. 2025 Oct 9;20(10):e0334019. doi: 10.1371/journal.pone.0334019 (PMC12510650; doi:10.1371/journal.pone.0334019)
Supplement: Attachment — Submitted filename: Response_to_Reviewers_auresp_3.docx [file pone.0334019.s011.docx]

Rebuttal Letter

Dear Editors and Reviewers,

We sincerely appreciate the time and effort you and the reviewers have dedicated to evaluating our manuscript. Your insightful comments have been invaluable in helping us improve the quality of our work. We have carefully considered all suggestions and implemented the necessary revisions as detailed below.

**In response to the Academic Editor's concern regarding references**, we deeply appreciate your careful review. Upon thorough re-examination, we regretfully identified that references 18, 19, and 20 could not be obtained through standard academic databases. To address this, we have removed these references and carefully revised the relevant sections. We have incorporated the study by Enzate T et al. as the new reference 18, which provides stronger support for our discussion. The content previously citing references 18 and 19 has been replaced by reference 8. All corresponding text in the Discussion section has been meticulously updated to reflect these changes.

**Regarding Reviewer #6's valuable suggestions on methodology**, we are truly grateful for the thoughtful comments concerning methodological rigor. Following this advice, we have modified the Methods section by adding a comprehensive Risk of Bias assessment, including detailed processes of evaluation and the complete content of ROB2. We believe this addition significantly strengthens the methodological framework of our study.

All modifications have been carefully highlighted in the revised manuscript for your convenience. We sincerely hope these revisions meet with your approval. Finally, we wish to express our deepest gratitude for your guidance throughout this review process. Your expertise has been instrumental in helping us improve our manuscript.

With warm regards and highest esteem,

Fahui Yin

Cardiovascular Department, Liangzhou Hospital

Wuwei City, Gansu Province, China 733000

Email：[414380580@qq.com](mailto:414380580@qq.com)

Tel: +86 13309359278
