# Supplementary material for: Updated meta-analysis of fractional flow reserve versus coronary angiography for guiding percutaneous coronary intervention
Source: PLoS One. 2025 Oct 9;20(10):e0334019. doi: 10.1371/journal.pone.0334019 (PMC12510650; doi:10.1371/journal.pone.0334019)
Supplement: Attachment — Submitted filename: Manuscript.docx [file pone.0334019.s012.docx]

#### Title Page

**Manuscript title:** Updated Meta-Analysis of Fractional Flow Reserve versus Coronary Angiography for Guiding Percutaneous Coronary Intervention.

**Short title:** Meta-analysis of FFR versus CAG guided PCI.

**Author list:** Fahui Yin^1*^MD; Yong Zhang^1^ MD; Xueqian Zhang^1^; Yangang Chen^1^MD; Xuelian Cui^1^;

**1** department of cardiovascular disease, Liangzhou Hospital, Wuwei City, Gansu Province, China

***Corresponding author:** Fahui Yin, MD, department of cardiovascular disease, Liangzhou Hospital, Wuli new village, Gaoba Town, Liangzhou District, Wuwei City, Gansu Province, China.

E-mail: 414380580@qq.com.

**Co-author:** Yong Zhang^1^ MD department of cardiovascular disease, Liangzhou Hospital, Wuli new village, Gaoba Town, Liangzhou District, Wuwei City, Gansu Province, China.

E-mail: zy8152@163.com.

**Word counts:** 2914 words.

**Updated Meta-Analysis of Fractional Flow Reserve versus Coronary Angiography for Guiding Percutaneous Coronary Intervention**

**Fahui Yin MD; Yong Zhang MD; Xueqian Zhang; Yangang Chen MD; Xuelian Cui;**

**Abstract**

**Background:** Fractional Flow Reserve (FFR) has been widely used in clinical practice for decades; however, the comparative clinical outcomes of FFR-guided versus coronary angiography (CAG)-guided percutaneous coronary intervention (PCI) require further comprehensive evaluation.

**Method:** Randomized controlled trials (RCTs) comparing FFR-guided and CAG-guided PCI were systematically searched across PubMed, Embase and the Cochrane library databases from their inception to December 31, 2023. The primary endpoints included the incidence of major adverse cardiovascular events (MACE), all cause mortality, myocardial infarction (MI) and target vessel revascularization(TVR). Stratified analyses were conducted to evaluate the effects of FFR-guided versus CAG-guided PCI across different follow-up periods (short-term and long-term) and patient categories (ACS and non-ACS patients).

**Results:** This meta-analysis included eight RCTs involving 4,433 patients, with four studies reporting 1-year outcomes and four reporting outcomes beyond one year. Among these, 5 studies focused on non-ACS patients, and three included ACS patients, with a significant male predominance (3,437 vs. 996 females). By follow-up duration, FFR-guided PCI demonstrated significant long-term reductions in MACE (OR: 0.76, 95% CI: 0.60-0.96, P = 0.022) and MI (OR: 0.65, 95% CI: 0.45-0.93, P = 0.018), but no significant short-term benefits were observed for MACE (OR: 0.85, 95% CI: 0.67-1.08, P = 0.194), MI (OR: 0.85, 95% CI: 0.63-1.16, P = 0.307), or all-cause mortality (short-term: OR: 0.77, 95% CI: 0.47-1.26, P = 0.296; long-term: OR: 0.74, 95% CI: 0.50-1.09, P = 0.123). By patient type, FFR-guided PCI significantly reduced MACE (OR: 0.82, 95% CI: 0.68-0.99, P = 0.038), MI (OR: 0.76, 95% CI: 0.58-0.99, P = 0.039), and TVR (OR: 0.78, 95% CI: 0.61-0.99, P = 0.036) in non-ACS patients, but no significant differences were observed in ACS patients for MACE (OR: 0.76, 95% CI: 0.53-1.08, P = 0.127), all-cause mortality (OR: 0.60, 95% CI: 0.35-1.02, P = 0.060), MI (OR: 0.77, 95% CI: 0.47-1.25, P = 0.294), or TVR (OR: 0.98, 95% CI: 0.48-2.02, P = 0.315). Sensitivity analysis confirmed the robustness of these findings.

**Conclusions:** FFR-guided PCI is superior to CAG-guided PCI in reducing MACE and MI in long-term and non-ACS patients but shows no advantage in short-term or ACS populations. Future research endeavors should focus on optimizing the application of FFR in ACS patients to potentially enhance clinical outcomes.

**Introduction**

Coronary artery disease (CAD) has been the leading cause of death worldwidely and exerts a severe health burden on human being internationally[1]. As an “golden standard” for diagnosis of CAD, coronary angiography (CAG) has always been the most important method for guiding PCI. However, owing to its nature of approximation, usually over-estimates or under-estimates the severity of the intermediate coronary stenosis, and the inability to determine the hemodynamic significance of coronary stenosis. Hence, the clinical importance of anatomical stenosis based on coronary angiography was challenged by FFR which could scientifically measure whether the target vessel have inducible ischemia through a special pressure guidewire[2].

FFR is defined as the ratio of the pressure distal to stenosis and the aortic pressure in a condition of maximum myocardium hyperemia. With a special coronary pressure wire, FFR could provide a readily available, straightforward and quantitative technique to evaluate the physiologic significance of a coronary lesion. When the value of FFR greater than 0.8, the stenosis is considered non-significant, and the value less than 0.75 is regarded as coronary ischemia[3]. FFR was recommended as Class IA for guiding percutaneous coronary revascularisation by European Society of Cardiology and class IIA by American Heart Association practice guideline[4,5].

Many studies have investigated the clinical outcomes of FFR-guided and CAG-guided PCI in patients with CAD. Fraction Flow Reserve Versus Angiography for Multivessel Evaluation (FAME) study showed FFR-guided PCI had lower rates of mortality, re-infarction and MACEs when compared to the standard angiography-guided PCI [6]. However, the DEFER-DES (Fractional Flow Reserve–Guided Versus Routine Drug-Eluting Stent Implantation in Patients With Intermediate Coronary Stenosis) trail, demonstrated that no significant difference was observed in cardiac death, revascularization and MACEs between the two groups with a five-year follow up[7]. Additionally，although previous meta-analysis have compared the two strategies, most of them enrolled the observational studies and the patients with different operation type (while some patients undergone PCI, others might select coronary artery bypass graft (CABG))[8,9]. ~~Consequently, we again compare the clinical outcomes of FFR-guided and CAG-guided PCI through even more rigorous quality control and patients selection.~~ In summary, previous studies presented mixed findings of clinical outcomes of FFR-guided and CAG-guided PCI in patients with CAD. Therefore, the aim of this study is to investigate the clinical outcomes of FFR-guided and CAG-guided PCI.

**Methods**

The Preferred Reporting Items for Systematic Reviews and Meta-Analyses (PRISMA) (Page et al., 2021) were used to guide the reporting of this meta-analysis. Elements of this quantitative method systematic literature review include Data sources and search strategy, Inclusion and exclusion criteria, Subgroup analysis, Data collection and extraction, Risk of bias assessment, and statistical analysis.

**Data sources and search strategy**

PubMed、Embase and the Cochrane library data bases were searched to identify articles comparing CAG and FFR-guided PCI before December 31,2023. All articles were restricted to English. According to the “PICOS” principle of evidence-based medicine, the following medical subject headings and search terms were used: (1) fractional flow reserve; (2) coronary angiography; (3) percutaneous coronary intervention. Additionally, references of relevant articles and conference reports were screened to uncover potential suitable studies. A comprehensive search strategy was developed after examination of titles and abstracts of related journal articles by using the search terms (Supplementary Material 1). ~~This study was carried out in accordance with the Preferred Reporting Items for Systematic Reviews and Meta-Analyses (PRISMA) statement.~~

**Inclusion and Exclusion criteria**

The PICO (Population, Intervention, Comparison, and Outcome) framework was used to formulate the research question. Studies were eligible for inclusion included if they met the following criteria: (1) Randomised controlled trails (RCTs) ; (2) Comparing FFR-guided and angiography-guided PCI; (3) Reporting at least one of the following outcomes, including MACEs, all-cause mortality, MI, and TVR. Studies were excluded if they were: (1) Non-RCTs; (2) Studies included the patients underwent CABG; (3) Did not report relevant outcomes; (4) Did not allow for the extraction of detailed data.

**Subgroup analysis**

In this meta-analysis, subgroup analysis were conducted based on the follow-up period and patients categories. Regarding the follow-up period, the studies were divided into short term subgroup (≤1 year) and long term follow-up subgroup(>1 year). In terms of patients categories, the studies were divided into acute coronary syndrome (ACS) subgroup and non-ACS subgroup.This type of subgroup analysis not only detect the potential heterogeneity among different studies, but also allow for a better comparison of the outcomes of FFR-guided and CAG-guided PCI in different patient populations and clinical backgrounds.

**Data collection and extraction**

Firstly, all records were imported into the Endnote software（version X7, Thomson Reuters, U.S）to perform automatic duplication removal during the initial search. After eliminating duplicates, three reviewers (YFH, ZY, and ZXQ) independently screened the titles and abstracts and excluded the irrelevant records. Subsequently, the full-text articles of the remaining records were retrieved for in-depth screening. Additionally, the reference of relevant articles and conference reports were also screened for potential suitable studies. A standard data extraction form was designed prior to data extraction. From each included study, the following information was extracted: the first author, year of publication, nationality, follow-up duration, sample size of each study, baseline characteristics, and the clinical outcomes (Please refer to Table ***). Any discrepancy in data collection and extraction were resolved through consensus.

**Risk of bias assessment**

The methodological quality of included randomized controlled trials was rigorously evaluated using the Revised Cochrane Risk of Bias tool (ROB 2.0) [10]. This comprehensive assessment examined five critical domains: (1) randomization process, (2) deviations from intended interventions, (3) missing outcome data, (4) measurement of outcomes, and (5) selection of reported results. Two independent reviewers conducted the evaluations using standardized criteria, with any discrepancies resolved through discussion until consensus was achieved.

**Statistical analysis**

Continuous variables were expressed as mean ± standard deviation (SD), while categorical variables were presented as numbers and percentages. Statistical analyses were performed using Stata software (version 18.0, Stata Corp, College Station, Texas). Heterogeneity among the included studies was assessed using the Chi-square test and quantified by the I² statistic. Given that all outcomes in this study were dichotomous data, they were pooled and expressed as odds ratios (ORs). A fixed-effect model (Mantel-Haenszel method) was applied when I² < 50%, indicating low heterogeneity, whereas a random-effect model (DerSimonian-Laird method) was used for I² ≥ 50%, indicating significant heterogeneity, with greater weight assigned to smaller studies. Subgroup analyses were conducted based on follow-up duration and patient categories to explore potential sources of heterogeneity and to compare fractional flow reserve (FFR) and coronary angiography (CAG) under different clinical scenarios. Sensitivity analysis was performed by comparing fixed- and random-effect models. Due to the limited number of included studies (fewer than ten), funnel plots were not utilized, as their power to detect asymmetry would be insufficient. Results were reported as ORs with 95% confidence intervals (CIs), and a p-value < 0.05 was considered statistically significant.

**Results**

**Searching results and quality assessment**

~~A comprehensive literature search was conducted in PubMed, Embase, and the Cochrane Library from their inception to December 31, 2023.~~The initial literature searching yielded 1959 articles, ~~which were independently screened by three team members (YFH, ZY, ZXQ) .~~ An additional 9 articles were identified through manual reference checking of the included studies. ~~First,~~ duplicates were removed through automated and manual deduplication processes. ~~Second,~~ after screening titles and abstracts, 29 articles were retained by excluding irrelevant ones (as detailed in S1 Table).. ~~Then, full text articles were obtained and carefully reviewed against predefined inclusion and exclusion criteria.~~ Ultimately, 8 studies met inclusion criteria and were enrolled into this meta-analysis[6,11-17]. The study selection process is illustrated in **Fig 1**. The revised Cochrane risk of bias (Rob2) tool was utilized to assess the quality of enrolled RCTs. Most studies described the detailed process of random sequence generation. However, since blinding of participants and intervention providers was not feasible, potential deviations from the intended interventions due to the experimental context were likely, though difficult to ascertain from the articles. Six studies employed independent clinical data and events committees to evaluate endpoints in a blinded manner. All studies provided detailed clinical endpoint events and accounted for missing data, but the source of these data (whether from multiple eligible outcome measurements) was unclear. The results of the risk of bias assessment are presented in  **S1 and S2 Figs.**

**Fig 1.** Flow Diagram of studies selection process.

**Baseline characteristics of the included studies**

Eight studies involving 4,433 patients that met our predefined inclusion criteria were included into this meta-analysis [6,11-17]. Among these, three studies compared FFR-guided and CAG-guided PCI in patients with multivessel patients [6,14,15]. two enrolled patients withSTEMI and multivessel patients [13,17], one focused on with older patients with NSTEMI [11], and the other two recruited patients with intermediate coronary stenosis and true coronary bifurcation lesions, respectively [7,12]. Baseline characteristics of the studies are presented in Table 1. The follow up duration was ranged from 1 year to 5 years, and the mean age ranged from 59.5 to 70 years. Male patients were significantly outnumbered female patients in overall population (3437 versus 996, respectively), with 24.5% having diabetes, 57.9% having hypertension, 56.3% having dyslipidemia, and 32.8% being current smokers. No significant difference was obsered between the two groups. Based on follow-up duration, the studies were further divided into short term ( ≤1 year) and long term (>1 year) follow-up groups: fourstudies with one-year follow-up belonged to the short term subgroup [6,11-13] , while the other four belonged to long term subgroup [14-17]. Aditionally, based on patients categories, the studies were divided into ACS [11,13,17] and non-ACS subgroups [6,12,14-16].

**Table1. Baseline characteristics of the included studies**

| Study | Quintella^14^ 2019 | | Puymirat^13^ 2021 | | Nunen^15^ 2015 | | Park^16^ 2015 | |
| --- | --- | --- | --- | --- | --- | --- | --- | --- |
| Follow-up (year) | 1.5 | | 1 | | 5 | | 5 | |
| Nationality* | Brazil | | France | | Netherland | | Korea | |
| Type of patients | MVD | | STEMI and MVD | | MVD | | ICS | |
| Group | CAG | FFR | CAG | FFR | CAG | FFR | CAG | FFR |
| Number of patients | 35 | 34 | 577 | 586 | 429 | 436 | 115 | 114 |
| Age, mean (SD),y | 60± 9 | 63 ± 8 | 62 ± 11 | 63 ± 11 | 64± 10 | 65 ± 10 | 63 ± 10 | 62± 10 |
| Male patients,No.(%) | 22 (63) | 25 (74) | 468 (81) | 498 (85) | 318 (74) | 328 (75) | 83(73) | 87(76) |
| Diabetes Mellitus,No.(%) | 12 (34) | 12 (35) | 82(14) | 107(18) | 107 (25) | 98 (22) | 39 (34) | 30 (26) |
| Hypertension, No.(%) | 26 (74) | 25 (74) | 262(45) | 253(43) | 277 (65) | 259 (59) | 73 (64) | 65 (57) |
| Dyslipedemia, No.(%) | 26 (74) | 24 (71) | 237(41) | 232(39) | 316 (74) | 307 (70) | 78 (68) | 80 (70) |
| Current smoker, No.(%) | 9 (26) | 10 (29) | 210(36) | 235(40) | 130 (30) | 111 (25) | 38 (33) | 30 (26) |
| Previous MI, No.(%) | 7 (20) | 8 (24) | 31(5) | 45(8) | 155 (36) | 154 (35) | 20 (17) | 22 (19) |
| Outcomes† | ①②④ | | ①②③④ | | ①②③④ | | ①④ | |
| Study | **Chen^12^ 2015** | | **Zhang^11^ 2016** | | **Tonino^6^ 2009** | | **Lee^17^ 2023** | |
| Follow-up (year) | 1 | | 1 | | 1 | | 3.5 | |
| Nationality* | China | | China | | Netherland | | Korea | |
| Type of patients | CAD with TCBL | | NSTEMI | | MVD | | STEMI and MVD | |
| Group | CAG | FFR | CAG | FFR | CAG | FFR | CAG | FFR |
| Number of patients | 160 | 160 | 110 | 110 | 496 | 509 | 278 | 284 |
| Age, mean (SD),y | 65 ± 9 | 65 ± 9 | 70 ± 3 | 70 ± 4 | 64 ± 10 | 65 ± 10 | 63 ± 12 | 64 ± 11 |
| Male patients, No.(%) | 116 (73) | 121 (76) | 78 (71) | 75 (68) | 360 (73) | 384 (75) | 234(84) | 240(85) |
| Diabetes Mellitus,No.(%) | 43 (27) | 48 (30) | 36 (33) | 40 (36) | 125 (25) | 123 (24) | 86(31) | 97(34) |
| Hypertension, No.(%) | 106 (68) | 116 (73) | 83 (76) | 81 (74) | 327 (66) | 312 (61) | 152(55) | 151(53) |
| Dyslipedemia, No.(%) | 32 (20) | 27 (17) | 93 (85) | 90 (82) | 362 (73) | 366 (72) | 107(39) | 121(43) |
| Current smoker, No.(%) | 64 (40) | 66 (41) | 31 (28) | 29 (26) | 156 (32) | 138 (27) | 105(38) | 91(32) |
| Previous MI, No.(%) | 19 (12) | 12 (8) | 24 (22) | 23 (21) | 180 (36) | 187 (37) | 7(3) | 7(3) |
| Outcomes† | ①②③④ | | ①②③ | | ①②③④ | | ①②③④ | |

Values are presented as percentage; “*” indicates the first author’s nationality; “†” four outcomes were included in this meta-analysis: ① indicates MACE; ②indicates all cause mortality; ③ indicates myocardial infarction; ④ targeted vessel revascularization.

MVD = multi-vessel disease; STEMI = ST segment elevation myocardial infarction; ICS = intermediate coronary stenosis; CAD = coronary artery disease; TCBL = true coronary bifurcation lesion.

**Clinical outcomes of FFR-guided and CAG-guided PCI**

**MACE.** A total of 8 studies involving 4433 patients reported MACE rates, with 4 studies providing 1-year outcomes[6,11-13] and the remaining 4 reporting outcomes beyond 1 year[14-17]. Stratified analysis based on follow up duration revealed no significant difference in short-term outcomes between the FFR and CAG groups (OR: 0.85, 95%CI 0.67-1.08, *P* = 0.194, *I^2^* = 35.9%). Sensitivity analysis confirmed the robustness of the results, as the pooled effect sizes calculated using both fixed-effect and random-effects models remained consistent. However, FFR-guided PCI demonstrated significant long-term benefits in reducing MACE rates compared to CAG-guided PCI  (OR:0.76, 95%CI 0.60- 0.96, *P* = 0.022, *I^2^* = 47.6% )(**Fig 2. A**). Among the included studies, 5 focused on non-ACS patients [6,12,14-16], while 3 included ACS patients [11,13,17]. Subgroup analysis by patient category showed that FFR-guided PCI was associated with greater reductions in MACE rates in the non-ACS subgroup( OR:0.82, 95%CI 0.68- 0.99, *P* = 0.038, *I^2^* = 0.0% ). no significant difference was observed in the ACS subgroup ( OR:0.76, 95%CI 0.53- 1.08, *P* = 0.127, *I^2^* = 76.9% )(**Fig 3. A**).

**All-cause Mortality.** When assessing all-cause mortality, 7 studies involving 4204 patients reported relevant data [6,11-15,17], including 4 studies with 1-year outcomes [6,11-13] and 3 studies with outcomes beyond 1 year [14,15,17]. Stratified analysis by follow-up duration revealed no significant differences in either the short-term (OR: 0.77, 95% CI: 0.47-1.26, P = 0.296, *I^2^* = 0%) or long-term groups (OR: 0.74, 95% CI: 0.50-1.09, P = 0.123, *I^2^* = 54.8%) (**Fig 2.B**).Further stratification by patient category showed that 4 studies focused on non-ACS patients [6,12,14,15], while 3 studies included ACS patients [11,13,17]. Subgroup analysis demonstrated no significant differences in all-cause mortality between FFR-guided and CAG-guided PCI, both in the ACS subgroup (OR: 0.60, 95% CI: 0.35-1.02, P = 0.060, *I^2^* = 32.8%) and the non-ACS subgroup (OR: 0.84, 95% CI: 0.58-1.21, P = 0.349, *I^2^* = 0%)(**Fig 3. B**). Sensitivity analysis confirmed the consistency of these findings, as the pooled effect sizes calculated using both fixed-effect and random-effects models remained stable across subgroups.

**Fig 2. Forest plot of subgroup analysis comparing FFR-guided versus CAG-guided PCI stratified by follow-up duration.**

**MI.** Regarding myocardial infarction, 6 studies involving 4135 patients reported MI incidence [6,11-13,15,17], including 4 studies with 1-year outcomes [6,11-13] and 2 studies with outcomes beyond 1 year [15,17]. Among these, 3 studies focused on non-ACS patients [6,12,15], while 3 included ACS patients [11,13,17]. Subgroup analysis revealed that FFR-guided PCI was associated with significantly fewer MI events compared to CAG-guided PCI in both the long-term group (OR: 0.65, 95% CI: 0.45-0.93, P = 0.018, *I^2^* = 71.9%) (**Fig 2 C**).and the non-ACS subgroup (OR: 0.76, 95% CI: 0.58-0.99, P = 0.039, *I^2^* = 0%)(**Fig 3 C**). However, no significant differences were observed in the short-term group (OR: 0.85, 95% CI: 0.63-1.16, P = 0.307, *I^2^* = 39.9%) or the ACS subgroup (OR: 0.77, 95% CI: 0.47-1.25, P = 0.294, *I^2^*= 77.1%) . The high I² value in the ACS subgroup suggested substantial heterogeneity. Upon reviewing the included studies, significant clinical heterogeneity was identified. First, the patient populations differed: Puymirat et al. [11] exclusively enrolled ST-segment elevation MI (STEMI) patients, Zhang et al. [13] included non-ST-segment elevation MI (NSTEMI) and elderly Chinese patients, while Lee et al. [17] enrolled both STEMI and NSTEMI patients. Second, the follow-up durations varied: Puymirat et al. [11] and Zhang et al. [13] followed patients for 1 year, whereas Lee et al. [17] extended follow-up to approximately 3.5 years.

**Fig 3. Forest plot of subgroup analysis comparing FFR-guided versus CAG-guided PCI stratified by patients categories**

**TVR.** TVR was reported in 7 studies involving 4213 participants. Subgroup analysis based on follow-up duration revealed no significant differences in TVR rates between FFR-guided and CAG-guided PCI, both in the short-term (OR: 0.75, 95% CI: 0.52-1.09, P = 0.196, *I^2^* = 48%) and long-term subgroups (OR: 0.82, 95% CI: 0.62-1.09, P = 0.243, *I^2^* = 0%) **(Fig 2 D)**. However, FFR-guided PCI was associated with significantly fewer revascularizations in the non-ACS subgroup (OR: 0.78, 95% CI: 0.61-0.99, P = 0.036, *I^2^* = 0%). In contrast, no significant difference was observed in the ACS subgroup (OR: 0.98, 95% CI: 0.48-2.02, P = 0.315, *I^2^* = 71.3%) **(Fig 3 D)**. Sensitivity analysis confirmed the stability and robustness of these outcomes.

**Discussion**

This study systematically compared the clinical outcomes of FFR-guided PCI versus CAG-guided PCI in terms of major adverse cardiovascular events, all-cause mortality, myocardial infarction , and target vessel revascularization through meta-analysis and stratified analysis. Overall, FFR-guided PCI demonstrated significant benefits in reducing MACE and MI rates, particularly in non-ACS patients and long-term follow-up, while no significant differences were observed in all-cause mortality. Subgroup analyses further highlighted the advantages of FFR-guided PCI in non-ACS patients.

FFR-guided PCI significantly reduced MACE rates in the long-term follow-up, but no significant difference was observed in the short-term follow-up. Subgroup analysis revealed that FFR-guided PCI significantly lowered MACE rates in non-ACS patients, while no significant difference was observed in ACS patients. A previous meta-analysis also support the superiority of FFR-guided PCI in reducing MACE[8]. However, the study did not differentiate between short-term and long-term outcomes or thoroughly analyze differences between ACS and non-ACS patients. Through stratified analysis, this study is first to clarify the long-term benefits of FFR-guided PCI and its advantages in non-ACS patients, addressing gaps in previous research.

No significant differences in all-cause mortality were observed between FFR-guided and CAG-guided PCI in either short-term or long-term follow-up. Subgroup analysis showed that FFR-guided PCI did not significantly reduce all-cause mortality in either ACS or non-ACS patients. This is consistent with Enezate T et al.study which also found no significant advantage of FFR-guided PCI in reducing all-cause mortality[18]. However, the study did stratified results by patient type. This study, through subgroup analysis, further confirmed the consistency of these findings in both ACS and non-ACS patients, providing more comprehensive evidence for the clinical application of FFR-guided PCI.

FFR-guided PCI demonstrated significant benefits in reducing MI rates during long-term follow-up, although no significant difference was observed in the short term. Subgroup analysis further revealed that this reduction in MI rates was particularly pronounced in non-ACS patients, while no significant benefit was observed in ACS patients. Similarly, FFR-guided PCI significantly decreased target vessel revascularization (TVR) events in non-ACS patients, but no such advantage was seen in ACS patients. Notably, subgroup analysis indicated that FFR-guided PCI did not significantly reduce TVR rates in either short-term or long-term follow-up across the overall population. These findings suggest that the benefits of FFR-guided PCI are more evident in non-ACS patients, likely due to the more stable pathophysiology of chronic coronary syndromes, which allows for more precise identification of ischemia-causing lesions. In contrast, the complex and dynamic nature of ACS, including plaque instability and thrombotic events, may limit the effectiveness of FFR-guided strategies in this population. The high heterogeneity observed in ACS subgroups further underscores the need for tailored approaches and standardized protocols in future studies to better define the role of FFR-guided PCI in ACS patients.

The long-term advantages of FFR-guided PCI in non-ACS patients likely stem from the stable pathophysiology of chronic coronary syndromes, enabling precise identification of ischemia-causing lesions and optimized revascularization strategies. By avoiding unnecessary interventions in non-ischemic lesions, FFR-guided PCI reduces procedural complications and late adverse events, such as stent thrombosis or restenosis, thereby improving long-term outcomes. In contrast, the lack of significant short-term benefits may reflect the time needed for clinical advantages to manifest, as early outcomes are often influenced by procedural factors rather than physiological assessment. In ACS patients, the complex and dynamic nature of the disease, including plaque instability, thrombus formation, and microvascular dysfunction, may limit the effectiveness of FFR-guided PCI . Physiological assessment in ACS can be confounded by acute changes, leading to less reliable measurements and suboptimal revascularization decisions. High heterogeneity in ACS subgroups, driven by variability in ACS subtypes (STEMI vs. NSTEMI) and FFR timing, further obscures its benefits.

Future studies should refine FFR-guided PCI in ACS patients by exploring optimal FFR timing, integrating intracoronary imaging (e.g., OCT or IVUS), and developing tailored strategies for different ACS subtypes. Additionally, long-term randomized controlled trials with standardized protocols are needed to validate FFR-guided PCI's benefits in non-ACS patients and clarify its role in ACS populations. Addressing these gaps will enhance FFR-guided PCI's precision and effectiveness across diverse patient groups, ultimately improving clinical outcomes.

**Innovations and Limitations.**

This study's innovations include a multidimensional stratified analysis by follow-up duration and patient type, providing comprehensive and precise clinical evidence, as well as comprehensive sensitivity analysis further verified the stability of the results, enhancing their credibility. However, several limitations should be noted. First, a significant gender imbalance was observed, with male patients predominating (3437 vs. 996), likely due to the higher prevalence of coronary heart disease in males, potentially introducing bias and limiting the generalizability of the findings. Second, due to the limited number of included studies (fewer than ten), funnel plot analysis for publication bias was not performed, as the test power would be insufficient to detect potential asymmetry. Additionally, to avoid duplication, two studies from the FAME trial (Nunen 2015 and Tonino 2009), reporting outcomes at different follow-up times, were analyzed separately through stratified analysis rather than pooled together. Addressing these limitations in future research will further strengthen the evidence base for FFR-guided PCI.

**Conclusions**

This meta-analysis demonstrates that FFR-guided PCI significantly reduces MACE and MI rates, particularly in non-ACS patients and long-term follow-up, but shows no significant benefits in ACS patients or short-term outcomes. Future research endeavors should focus on optimizing the application of FFR in ACS patients to potentially enhance clinical outcomes.

**Disclosure**

No potential conflicts of interest or financial support were reported for this review

**Reference:**

1. Mathers CD, Loncar D. Projections of global mortality and burden of disease from 2002 to 2030. PLoS Med 3(11): e442. [doi:org/10.1371/journal.](https://doi.org/10.1371/journal.)pmed.0030442.

2. [Kern MJ,](https://www.ahajournals.org/doi/10.1161/CIRCULATIONAHA.106.177276#con1) [Lerman A,](https://www.ahajournals.org/doi/10.1161/CIRCULATIONAHA.106.177276#con2) [Bech JW,](https://www.ahajournals.org/doi/10.1161/CIRCULATIONAHA.106.177276#con3) [Bruyne BD,](https://www.ahajournals.org/doi/10.1161/CIRCULATIONAHA.106.177276#con4) [Eeckhout E,](https://www.ahajournals.org/doi/10.1161/CIRCULATIONAHA.106.177276#con5) [Fearon WF](https://www.ahajournals.org/doi/10.1161/CIRCULATIONAHA.106.177276#con6) et al. [Physiological Assessment of Coronary Artery Disease in the Cardiac Catheterization Laboratory: A Scientific Statement From the American Heart Association Committee on Diagnostic and Interventional Cardiac Catheterization, Council on Clinical Cardiology](http://pubmed.ncbi.nlm.nih.gov/16940193/).Circulation 2006. 114:1321–1341. doi: 10.1161/circulationaha.106.177276.

3. Watkins S, McGeoch R, Lyne J, Steedman T, Good R, McLaughlin MJ et al. Validation of magnetic resonance myocardial perfusion imaging with fractional flow reserve for the detection of significant coronary heart disease. Circulation. 2009;120(22):2207–13. doi:10.1161/circulationaha.109. 872358.

4. Neumann FJ, Sousa-Uva M, Ahlsson A, Alfonso F, Banning AP, Benedetto U et al. 2018 ESC/EACTS Guidelines on myocardial revascularization. The Task Force on myocardial revascularization of the European Society of Cardiology (ESC) and European Association for Cardio-Thoracic Surgery (EACTS). European Heart Journal (2019) 40, 87–165. doi:10.1093/eurheartj/ehy394.

5. Levine GN, Bates ER, Blankenship JC, Bailey SR, Bittl JA, Cercek B et al. 2011 ACCF/AHA/SCAI Guideline for Percutaneous Coronary Intervention. A report of the American College of Cardiology Foundation/American Heart Association Task Force on Practice Guidelines and the Society for Cardiovascular Angiography and Interventions. J Am Coll Cardiol 2011;58: e44–122. doi:10.1016/j.jacc.2011.08.007.

6. Tonino PA, Bruyne BD, Pijls NH, Siebert U, Ikeno F, Veer M et al. Fractional Flow Reserve versus Angiography for Guiding Percutaneous Coronary Intervention. N Engl J Med 2009; 360:213-24. doi:10.1056/NEJMoa 0807611.

7. Park SH, Jeon KH, Lee JM, Nam CW, Doh JH, Lee BK et al. Long-term clinical outcomes of fractional flow reserve-guided versus routine drug-eluting Stent Implantation in patients with intermediate coronary stenosis: Five-year clinical outcomes of DEFER-DES Trial. Circ Cardiovasc Interv. 2015;8(12):e002442. doi:10.1161/circinterventions.115.002442.

8. Zhang DF, Lv SZ, Song XT, Yuan F, Xu F,Zhang M et al. Fractional flow reserve versus angiography for guiding percutaneous coronary intervention: a meta-analysis. Heart 2015; 101:455-62. doi:10.1136/heartjnl-2014-306578.

9. Bundhun PK, Yanamala CM, Huang F. Comparing the adverse clinical outcomes associated with fraction flow reserve-guided versus angiography-guided percutaneous coronary intervention: a systematic review and meta-analysis of randomized controlled trials.BMC Cardiovascular Disorders (2016) 16:249. doi:10.1186/s12872 -016-0427-8.

10. Julian PT, Altman DG, [Gtzsche](https://xueshu.baidu.com/s?wd=author:(PC%20Gtzsche)%20&tn=SE_baiduxueshu_c1gjeupa&ie=utf-8&sc_f_para=sc_hilight=person) PC, [Jüni](https://xueshu.baidu.com/s?wd=author:(P%20J%C3%BCni)%20&tn=SE_baiduxueshu_c1gjeupa&ie=utf-8&sc_f_para=sc_hilight=person) P, [Moher](https://xueshu.baidu.com/s?wd=author:(D%20Moher)%20&tn=SE_baiduxueshu_c1gjeupa&ie=utf-8&sc_f_para=sc_hilight=person) D, [Oxman](https://xueshu.baidu.com/s?wd=author:(AD%20Oxman)%20&tn=SE_baiduxueshu_c1gjeupa&ie=utf-8&sc_f_para=sc_hilight=person) AD et al. The Cochrane Collaboration’s tool for assessing risk of bias in randomized trials. BMJ 2011;343: d5928. doi:10.1136/bmj. d5928.

11. Zhao Zhang, Ke Li., Jinwen Tian. Efficacy and safety outcomes of fractional flow reserve in guiding clinical therapy of non-ST-segment elevation myocardial infarction compared with angiography alone in elderly Chinese patients. Clinical Interventions in Aging 2016; 11:1751–1754. doi:org/10.2147/CIA.S123735.

12. Chen SL, Ye F, Zhang JJ, Xu T, Tian NL, Liu ZZ et al. Randomized Comparison of FFR-Guided and Angiography-Guided Provisional Stenting of True Coronary Bifurcation Lesions. JACC: cardiovascular interventions 2015; 8(4) :536-546. doi.org/10.1016/ j.jcin.2014.12.221.

13. Puymirat E, Cayla G, Simon T, Steg PG, Montalescot G, Durand-Zaleski I et al. Multivessel PCI Guided by FFR or Angiography for Myocardial Infarction. N Engl J Med 2021; 385(4):297-308. doi:10.1056/NEJMoa2104650.

14. Quintella EF, Ferreira E, Azevedo VM, Araujo DV, Sant`Anna FM, Amorim B et al. Clinical Outcomes and Cost-Effectiveness Analysis of FFR Compared with Angiography in Multivessel Disease Patient. Arquivos Brasileiros de Cardiologia 2019; 112(1):40-47. doi:10.5935/abc.20180262.

15. van Nunen LX, Zimmermann FM, Tonino PA, arbato E, Baumbach A, Engstrøm T et al.Fractional flow reserve versus angiography for guidance of PCI in patients with multivessel coronary artery disease (FAME):5-year follow-up of a randomised controlled trial. Lancet 2015; 386 (10006):1853-1860. doi:org/10.1016/S0140- 6736(15)00057-4.

16. Park SH; Jeon KH; Lee JM,Nam CW, Doh JH, Lee BK et al. Long-Term Clinical Outcomes of Fractional Flow Reserve Guided Versus Routine Drug-Eluting Stent Implantation in Patients With Intermediate Coronary Stenosis Five-Year Clinical Outcomes of DEFER-DES Trial. Circ Cardiovasc Interv. 2015;8:e002442. doi:10.1161/circinterventions. 115.002442.

17.Lee JM, Kim HK, Park KH, Choo EH, Kim CJ, Lee SH et al. Fractional flow reserve versus angiography-guided strategy in acute myocardial infarction with multivessel disease: a randomized trial. European Heart Journal (2023) 44, 473–484. doi: 10.1093/eurheartj/ehac763.

18. Enezate T, Omran J, AI-Dadah AS, Alpert M, White CJ, Abu-Fadel M et al. Fractional flow reserve versus angiography guided percutaneous coronary intervention: An updated systematic review. Catheter Cardiovasc Interv. 2017;1–10. doi: 10.1002/ccd.27302.

**Supplementary Online Content**

**S1 Table.** Sensitivity analysis of comparing different effect model

**S1 Fig.** Review authors' judgements about each risk of bias item presented as percentages across all included studies.

**S2 Fig.** Risk of bias summary: review authors' judgements about each risk of bias item for each included study.
