# Supplementary material for: Updated meta-analysis of fractional flow reserve versus coronary angiography for guiding percutaneous coronary intervention
Source: PLoS One. 2025 Oct 9;20(10):e0334019. doi: 10.1371/journal.pone.0334019 (PMC12510650; doi:10.1371/journal.pone.0334019)
Supplement: Attachment — Submitted filename: Response_to_Reviewers_auresp_4.docx [file pone.0334019.s013.docx]

**Rebuttle letter**

Dear Haipeng Liu Editor and Respected Reviewers:

We are truly grateful to the Editor for coordinating the review process and providing clear directions for our revision, which has helped us navigate the improvement of the manuscript efficiently. We also wish to extend our sincere thanks to each Reviewer for devoting time and expertise to evaluate our work—your detailed comments and insightful suggestions have not only pointed out key areas for optimization but also deepened our understanding of the research’s presentation and academic implications.

After a thorough discussion of all feedback from the Editor and Reviewers, we have addressed every comment one by one, making targeted revisions to the manuscript (e.g.Revising the plot for the risk of bias assessment using R software with the 'robvis' package, adding a table of search items, filters, and results, polishing the language in the manuscript, and so on.). In the following sections of this letter, we will respond to each point specifically, linking the feedback to the corresponding changes in the revised manuscript for clarity.

**For the reviewer #7:**

**Question 1:** In the introduction section,While explaining the rationale for the study, the authors write ‘Consequently, we again compare the clinical outcomes of FFR-guided and CAG-guided PCI through even more rigorous quality control and patients’ selection’ The authors should additionally consider documenting how pooling of randomized data at a large scale would give more clinically relevant findings than non-randomized data.

**Response:** We sincerely thank you for this key comment. From the perspective of methodology, randomized controlled studies provide more objective and convincing results than non-randomized studies by employing randomization and blinding procedures.However, conducting RCTs in daily practice is more challenging, especially when implementing blinding procedures for patients, investigators, and other relevant personnel. This is the fundamental reason why RCTs are regarded as providing the highest level of evidence in clinical research. In previous meta-analyses or studies regarding the comparison of CAG and FFR in guiding PCI, most were derived from observational studies. With the accumulation of experience and practice, we can now conduct meta-analyses based on high-level RCT evidence.

**Question 2:** In methods section,the authors should make a table for the search strategy used for each database and in the next column, specify the number of results that was obtained after the search string was run. This table should be added in supplementary files and referenced in the main manuscript appropriately. This would ensure reproducibility at a broader level.

**Response:** Based on your suggestions, we have created a table including the following columns: database, filter, time, and results. This table has been uploaded as a supplementary file.

**Questions 3:** From what I know, it is generally advised to use relative risk as an effect size for meta-analysis when all the included studies are RCTs. The authors have used OR instead. Authors should consider giving an explanation for why OR was used. If they decide to switch to RR instead, it should also be explained while being backed up by some strong references.

**Response:** We appreciate the reviewer’s valuable comment regarding the choice of effect measures. In our meta-analysis, we opted for odds ratios (ORs) rather than relative risks (RRs) for the following reasons:1.ORs are the most widely reported effect measure in cardiovascular intervention studies, particularly in studies comparing FFR- vs. angiography-guided PCI. 2.ORs are mathematically stable in both fixed- and random-effects models, especially when event rates vary across studies. In contrast, RRs can overestimate effects when outcome rates are high (>10%), which might be the case in some PCI trials. 3.While RRs may offer a more straightforward clinical interpretation, ORs are well-established in meta-analyses of binary outcomes, particularly in cardiology research. Importantly, when event rates are low (<10%), ORs approximate RRs, minimizing practical differences in interpretation.

**Question 4:** Explain more from what authors mean by non-ACS? It’s a vague term and demands more explanation. Does it mean Stable CAD patients?

**Response:** We appreciate the reviewer's insightful comment regarding the terminology. The term 'non-ACS' in our study specifically refers to patients with stable CAD manifestations, including those with: (1) multivessel disease, (2) intermediate coronary stenosis, and (3) true coronary bifurcation lesions. We have now clarified this definition in the revised Subgroup Analysis section to avoid any ambiguity.

**Question 5:** Results:It would be better if the results of the ROB 2.0 tool can be visually depicted using the Robvis tool. In supplementary files the authors call the traffic light plots ‘ Review of authors’ judgement’ which, theoretically is not wrong, but is a non-formal way of presentation. The authors should consider changing it to ‘Traffic Light Plots for Risk of Bias Assessment for Randomized Controlled Trials or something like this. Additionally, the current plots are from Rob 2 macros, which is obsolete now. Try making them on Robvis which gives clearer and more appropriate images. Make sure to cite the tool.

Response: According to your advice, we have used R software with packages like ‘robvis’ and ‘ggplot2’ to revise the risk of bias assessment plot. Meanwhile, the name of the plot has been revised (see Supplemental materials).

**Question 6:** For the flow diagram, instead of using the self-made flowchart, trying using PRISMA 2020 version and customize it as per study needs.

**Response:** Based on your suggestion, we have revised the flow diagram using PRISMA 2020 version (as described in the manuscript).

**Question 7:** Discussion:In the limitations section, the authors have detailed multiple methodological limitations. The authors should focus more on clinical limitations. Heterogeneity, even if mild for some outcomes, should be mentioned. How pooling ACS and Non-ACS patients together negatively impacts clinical interpretation albeit the subgroup analysis.Additionally, there are similar meta-analysis published in literature. The authors should discuss their findings in light of these already published meta-analyses. Their questions might be little bit different but they are definitely relevant here. Some I could find are: PMID: 25637372, 36115726.The authors may want to discuss if there may be an effect of underlying medications on the pooled results? Is it possible that some medications that these patients may have been currently on, lead to changes in reported parameters?

**Response:** As you pointed out, in the limitations section, we initially only listed limitations from a methodological perspective. Following your advice, we have revised this section to include limitations from a clinical standpoint as well. However, certain limitations could not be removed, as these were previously raised by reviewers. Additionally, we have refined the discussion section by incorporating insights from previously published meta-analyses. These suggestions have been extremely helpful. Regarding the impact of medication on clinical outcomes, we have addressed this point in the limitations section.

**Question 8:** Rebuttal Letter:The authors should give a point-to-point response to each comment by the reviewer, with page and line numbers detailed for each change done. Right now, the authors’ responses are vague, generic and difficult to follow.

Response: Thank you for your suggestion. We have made a point-to-point response to each comment, and each revision has been accompanied by the detailed page and line numbers.

**Question 9:T**he PRISMA checklist is missing. It’s a basic requirement for any Systematic review or meta-analysis and it is considered incomplete without it, as per the Cochrane guidelines. It should be added to supplementary files. A graphical abstract would be a nice addition.

**Response:** Thank you for your suggestion, actually, we have uploaded the PRISMA checklist in supplement materials(as uploaded in supplement files). we have revised the checklist according to the manuscript.

**For the Reviewer #8:**

**Question 1:** The manuscript would benefit from moderate language polishing to improve clarity and readability.

**Response:** Thank you for your helpful suggestions. We highly value this comment and have promptly acted on it. We have carefully revised the manuscript’s language, including adjusting awkward sentence structures, clarifying vague descriptions, and optimizing the logical connection of content to ensure that our research is presented more clearly. These revisions aim to make the manuscript easier for readers to comprehend, and we believe they have effectively addressed your concern.

**Question 2:** For outcomes like MI in ACS patients, heterogeneity was high (e.g., I² = 77%). It would strengthen the discussion to briefly address potential sources (e.g., STEMI vs NSTEMI, timing of FFR during acute phases, variability in clinical endpoints).

**Response:** Tank you for your advice, we have discussed the potential reason of high I^2^ in the Results section (Page 14 line 183-189).

**Question 3:** The authors recommend routine use of FFR-guided PCI across clinical practice. Given the null findings in ACS and short-term follow-up, I would suggest softening the language slightly to better reflect the stratified results. Acknowledging that the strongest benefit is in non-ACS and long-term scenarios would improve alignment with the data.

**Response:** Thank you sincerely for your insightful and constructive suggestions, which have been invaluable in refining the quality of our manuscript. Following your guidance, we have thoroughly revised the content in the Discussion and Conclusion sections. Specifically, we have further emphasized the effectiveness of FFR-guided PCI in patients with non-ACS and the long-term subgroup, clarifying the clinical relevance of these findings as you kindly highlighted. Additionally, we have supplemented our recommendation to avoid the use of FFR in ACS patients, ensuring this key point is presented with greater clarity and alignment with your feedback.

**Question 4:** Consider ensuring uniform phrasing for “short-term,” “long-term,” “non-ACS,” etc., throughout the manuscript. Some inconsistencies were noted in hyphenation and formatting.

**Response:** Following your valuable advice, we have conducted a thorough review of these phrases throughout the manuscript, ensuring consistent expression, uniform hyphenation, and standardized formatting.

We sincerely appreciate the reviewer’s thoughtful and constructive comments, which have been invaluable in guiding us to refine our study.This manuscript has now undergone five rounds of revisions, and we have carefully incorporated all feedback to ensure better alignment with the journal’s high standards. We earnestly hope this revised version will meet the requirements for successful review. Should any further adjustments or additional details be needed, we ready to respond promptly and thoroughly.

Sincerely,

Fahui Yin

Cardiovascular department, Wuwei Liangzhou Hospital

Wuwei City, Gansu Province,China

E-mail:414380580@qq.com
